# Likes, lies, and oversight: problematic social media use, concealment, parental monitoring, and adolescent cyberbullying

**DOI:** 10.3389/frcha.2026.1933453

**Published:** 2026-10-28

**Authors:** Megan Gale, Katherine N. Alexander, Sarah E. Schwartz, Diana J. Meter, Sarah M. Coyne, Drew P. Cingel

**Affiliations:** 1 Department of Human Development and Family Studies, Utah State University, Logan, UT, United States; 2 School of Family Life, Brigham Young University, Provo, UT, United States; 3 Department of Communication, University of California, Davis, CA, United States

**Keywords:** cyberbullying, generalized linear mixed modeling, school-level influence, social media use, youth risk behavior

## Abstract

**Introduction:**

The rapid expansion of social media has created new opportunities for adolescent connection but has also heightened risks of involvement in online aggression and cyberbullying. This study examined how problematic social media use, social media concealment, and parental media monitoring relate to cyberbullying victimization and perpetration among adolescents.

**Methods:**

Participants include 686 adolescents ages 12–18 from 12 schools. Logistic generalized linear mixed models were used to account for the nested structure of students within schools.

**Results:**

Results indicated that both problematic social media use and concealment from parents were significantly associated with greater odds of cyberbullying victimization and perpetration, whereas parental media monitoring was not a significant predictor in either model.

**Discussion:**

Findings highlight the importance of addressing adolescents’ specific patterns of media use and point to multi-systemic prevention approaches involving families, schools, and technology developers to mitigate cyberbullying risks.

## Introduction

The expansion of social media accessibility and availability has created new social opportunities, but it has also introduced social risks related to online harassment and aggression. Cyberbullying is a particularly pressing concern as social media use continues to rise among younger generations (1). Nearly one-third to one-half of adolescents in the United States have been victims of cyberbullying (2, 3), underscoring the scope of this issue. Given the ubiquity of social media use among adolescents (4, 5), there is a critical need to better understand the factors that place youth at greater risk for cyberbullying involvement. Although definitions vary somewhat across studies, cyberaggression refers broadly to intentional harm delivered through digital media, whereas cyberbullying typically involves repeated harmful behavior that includes a power imbalance between perpetrator and victim (6). Because the present study addresses adolescents’ experiences of harmful online interactions without directly measuring repetition or power imbalance, our outcome is conceptually more consistent with cyberaggression. However, researchers often use these terms interchangeably and the behaviors examined closely parallel those studied in cyberbullying research (6), we use the term cyberbullying throughout while acknowledging this distinction. In the present study, the available indicators capture verbal forms of online aggression through mean or hurtful comments.

Prior research has established that cyberbullying involvement is associated with significant risks for adolescent well-being, yet less is known about how specific patterns of social media use and family processes jointly contribute to these experiences. Cyberbullying is commonly conceptualized in terms of victimization and perpetration. *Cyberbullying victimization* specifically refers to being the recipient or victim of aggressive online behavior, and *cyberbullying perpetration* refers to committing acts of hostile online behavior (7). These experiences are associated with a range of profound negative outcomes. Cyberbullying victimization is associated with increased depression, anxiety, loneliness, somatic symptoms (8, 9), and psychological distress (10, 11), and adolescent cyber-victims are 50% more likely to self-harm and to experience suicidal ideation compared to their peers who are not cyberbullied (10, 12, 13). Additionally, cyberbullying perpetrators report greater substance use, direct peer aggression, and delinquent behaviors (8). Despite well-documented links between cyberbullying and adolescent mental health, considerably less is known about how specific patterns of adolescents’ social media use and family processes jointly contribute to cyberbullying victimization and perpetration. Recent work has increasingly examined qualitative and behavioral dimensions of social media use, including the purpose and type of use (14, 15), active versus passive use (16, 17), and content consumption and production (4, 18). However, less is known about how specific maladaptive patterns of use and adolescents’ management of their online behaviors within the family context jointly relate to cyberbullying. In particular, social media concealment (hiding or misrepresenting online behaviors, often from parents) remains comparatively understudied, and little is known about how concealment and problematic social media use (compulsive or maladaptive patterns of engagement with social media) relate to both cyberbullying victimization and perpetration when considered alongside parental media monitoring (parental efforts to track, guide, or co-use media with their children). The present study addresses this gap by examining associations between problematic social media use, social media concealment, and parental media monitoring and cyberbullying victimization and perpetration among adolescents.

This study draws on Bronfenbrenner's Process-Person-Context-Time (PPCT) model (19) to examine how adolescent behaviors, parental involvement, and school contexts relate to cyberbullying victimization and perpetration. Given the centrality of digital media in adolescent life, we also incorporate the Ecological Techno-Subsystem (20, 21), which extends the ecological model to include digital technologies as embedded, interactive elements of adolescents’ immediate environments.

*Proximal processes* are enduring, reciprocal interactions that drive development, such as those between adolescents and their peers, parents, or media. Cyberbullying functions as a proximal process when adolescents engage in harmful online interactions. Problematic social media use and concealment from parents, as well as parental monitoring strategies (e.g., restrictive, active, or co-use), all represent proximal processes that influence how adolescents navigate and respond to digital aggression (22, 23).

The *person* component reflects individual characteristics that shape, and are shaped by, development. Adolescents differ in how they engage with digital platforms: some may use social media for connection and self-expression (24), while others engage in more risky or aggressive behaviors (25). In cyberbullying, personal tendencies related to media use, may heighten vulnerability to both victimization and perpetration. These person-level differences, including tendencies toward problematic use or secrecy, interact with broader social contexts and digital environments to influence cyberbullying involvement. Additionally, research consistently demonstrates sex differences in both social media use patterns and cyberbullying involvement (1, 26, 53). Additionally, given developmental differences in social media and cyberbullying involvement across adolescence, age was included alongside biological sex assigned at birth as a covariate in the analyses.

Development occurs in *context*, or nested within ecological systems. Family and school microsystems are especially relevant to adolescent cyberbullying risk. Parents influence youth media habits through monitoring and communication (23), while schools contribute through peer group norms, media-related policies, and anti-bullying efforts (27). Additionally, the techno-subsystem situates digital environments (e.g., social media platforms) within these core contexts, emphasizing that online interactions are embedded in daily relationships and institutional cultures (20, 21). Peer attitudes endorsing aggression (22), school bullying experiences (6), and broader platform norms all shape cyberbullying experiences.

Adolescence is a developmentally sensitive *time* marked by increased social media engagement, peer influence, and identity exploration. As digital norms and platforms continue to evolve, so do the forms and meanings of cyberbullying. Shifting societal attitudes, historical context, and the timing of media exposure across adolescence all contribute to the ways that adolescents experience cyberbullying today (4).

For many years, researchers have investigated how time spent on social media was associated with cyberbullying (10). More recent research has expanded beyond time-based measures to examine qualitative and behavioral dimensions of social media use, including different patterns and forms of engagement (14, 15). This work increasingly suggests that understanding *how* adolescents utilize social media, in addition to how much they use it, is important for understanding cyberbullying (28).

Adolescents encounter various risk factors for cyberbullying when engaging with social media, and research examining different patterns of use suggests that problematic social media use may be particularly relevant to cyberbullying involvement (1, 29, 30). Previous research has included factors such as preoccupation with social media, negative feelings and psychological effects when not accessing social media, and negative real-life consequences because of excessive use, including mood changes (28). Although definitions vary, problematic social media use typically includes addiction-like symptoms such as compulsive checking, emotional dependency, interpersonal conflict, and neglect of offline responsibilities (31).

Estimates regarding problematic social media use vary widely depending on measurement and cutoffs. Earlier research suggested that about 4.5% of youth engaged in problematic social media use (32), but more recent work places prevalence substantially higher, with estimates varying depending on measurement approach. Recent estimates suggest that approximately 11% of adolescents worldwide exhibit problematic social media use, with prevalence estimates as high as 40% among U.S. adolescents (33, 34). Across multiple international samples of adolescents, researchers have found that youth who experience problematic patterns of media use are significantly more likely to be involved in cyberbullying perpetration and victimization (1). Problematic media use may heighten cyberbullying risk by increasing interpersonal conflict, emotional dysregulation, and social isolation.

These associations may operate through several mechanisms. Adolescents with problematic use spend more time online, increasing exposure to both risks and opportunities for cyber aggression. However, time alone does not explain the link. Problematic use also predicts heightened emotional dependency on social media, greater interpersonal conflict, impaired self-regulation, poorer emotion regulation, and more impulsive online behavior, all of which may increase both the likelihood of engaging in hostile behavior and the vulnerability to victimization in digital spaces (28–30). It is also important to note that peer problems, including cyberbullying victimization, may precede problematic social media use (35). This suggests a potentially cyclical process, where adolescents with existing peer difficulties turn to social media in maladaptive ways, which increases conflict, emotional strain, and the likelihood of further victimization or perpetration.

Adolescents also differ in their willingness to discuss social media use with others. *Social media concealment* is a seldom-studied construct that refers to intentionally hiding or misrepresenting online activities when communicating with others, often parents (36). Adolescents who are more transparent with their parents about their media use tend to exhibit greater prosocial behavior, whereas media secrecy is linked to lower prosocial tendencies and higher relational aggression. Adolescents who frequently conceal media use may be more likely to engage in in-person relational aggression (36). To our knowledge, there is no previous research assessing the association between social media concealment and cyberbullying victimization or perpetration. Investigating concealment is important because secrecy in media behaviors may undermine parental monitoring, reduce opportunities for guidance, and increase unsupervised exposure to risky online interactions, making it a potentially important but overlooked factor in cyberbullying involvement.

Social contexts play a role in shaping social media use patterns and cyberbullying risks. Family processes may be particularly important because parents shape adolescents’ digital environments through communication, supervision, and guidance. According to ecological and techno-subsystem perspectives (19, 20), parents remain influential even as peer interactions increasingly occur online. Within family contexts, parents establish media rules, discuss online safety, and monitor their children's digital engagement. Effective parental involvement may reduce adolescents’ exposure to risky online interactions by encouraging healthy digital habits and establishing appropriate boundaries around media use. Parent involvement through media monitoring has shown to buffer against the negative effects of social media use (37) and cyberbullying risks (23, 38). Consequently, parental media monitoring may help explain variation in cyberbullying involvement beyond adolescents’ own media behaviors.

Furthermore, *school contexts* shape cyberbullying, yet few studies have investigated how this nested component is related to such risks (22). There is a common misconception that strangers are most likely to perpetuate cyberbullying; in reality, perpetrators often include peers already known to the target, especially from shared school contexts (26). More broadly, this setting is one of the most salient contexts in adolescents’ lives (39). School-related social agents become important as children transition into adolescence and they spend substantially more time with peers, teachers, and non-family supports compared to family members (40). Adolescents rely on peers for implicit social norms related to media and technology usage, and schools may elect to have more explicit policies around social media usage, technology use, and how to handle cyberbullying (27). In sum, it is likely that school-based factors shape cyberbullying experiences, so there is a need to consider school as a higher-level nesting variable when accounting for these types of events (41).

The aim of the current study is to investigate the relationship between problematic social media use, social media concealment, parental media monitoring, and cyberbullying risks (victimization and perpetration). Given the role that schools play in shaping experiences, logistic generalized linear mixed models were utilized to account for the nested structure of participants in schools. We hypothesized that greater problematic social media use and greater social media concealment would be associated with increased odds of both cyberbullying victimization and perpetration, whereas greater parental media monitoring would be associated with decreased odds of both cyberbullying victimization and perpetration.

## Methods

### Sample and procedure

Data for this project were obtained from a larger research project examining social media use and government social media policy for adolescents, conducted by a collaborative multi-university research team. All methods for this study were approved by the Institutional Review Board of Brigham Young University (IRB2023-324). Schools were recruited through a multistage, nonprobability sampling process. Nine school districts were approached to participate in the study, with five districts agreeing to participate. Thus, district and school participation was based on willingness to participate rather than random selection. Administrators at each middle, junior high, and high school within participating districts were then contacted, with 12 schools agreeing to participate. Within participating schools, an email was sent to all parents inviting them to provide consent for their adolescent to participate in a study on social media and mental health; participation was therefore not restricted to particular grades or classes. Thus, the sample was drawn from multiple school contexts but was not designed to be representative of all adolescents in the state. After receiving parental consent and adolescent assent, a team of research assistants tested each adolescent in person at school during a prep period or free time. Participants completed a short online survey and research assistants were on site to answer questions or clarify any piece of the research. Testing time took approximately 20 min and participants were paid $10 for their time.

The initial dataset included 1,029 individuals who accessed the study. Of these 1,024 responded to the item assessing whether they used social media, with 693 reporting social media use. Participants who reported that they did not use social media (*n* = 331) were routed past the social-media-specific portion of the survey and therefore did not receive the measures examined in the present study. Five participants did not respond to the social media use item. Among the 693 adolescents who reported using social media, item-level missingness on the focal measures was low. Seven additional participants were excluded during data cleaning because they lacked sufficient data to derive the cyberbullying outcome variables; two of these participants also met the broader missing-data exclusion criteria because all focal analysis variables were missing. The final analytic sample included 686 adolescents ages 12–18 years old (*M* = 15.22, *SD* =1.55). Patterns of survey completion did not significantly differ across the 12 participating schools, providing no evidence of systematic differential attrition by school.

In the final analytic sample, 278 (40.8%) participants identified as male at birth, and 404 (59.2%) identified as female at birth. Regarding race and ethnicity, 537 participants (78.7%) identified as White and 145 (21.3%) identified with a minoritized racial or ethnic group (including 6% Hispanic/Latinx and 3.5% multiracial). These proportions are comparable to statewide demographic estimates (*Data USA*, n.d.). See Supplementary Table S1 in the supplemental material for descriptive statistics grouped by school.

As a sensitivity analysis, models were additionally adjusted for race and perceived family financial resources among the 555 adolescents with complete data on these covariates. Primary models were first re-estimated in this same subsample to distinguish changes associated with covariate adjustment from those resulting from case deletion. Full results are provided in Supplementary Table S3 in the supplemental material.

This study was not preregistered and data are not made publicly available.

### Measures

*Problematic social media use* (31) was assessed using seven items reflecting maladaptive or compulsive patterns of social media engagement, including emotional dependence, preoccupation, interference with other activities and relationships, and difficulty regulating use. Items were scored from l (*Strongly Disagree*) to 5 (*Strongly Agree*) and averaged to create a total score. Sample items include “It would be very difficult, emotionally, to give up social media,” “The amount of time I spend using social media keeps me from doing other important things,” and “When I am not using social media, I am thinking about using it or planning the next time I can use it.” Importantly, none of the items directly assess cyberbullying behaviors. The scale demonstrated acceptable internal consistency with a Cronbach's alpha of.76.

*Social media concealment and disclosure* (36) was assessed using four items reflecting adolescents’ intentional withholding or disclosure of information about their social media use to parents. Items captured concealment of social media content and frequency of use, as well as communication with parents about media use. Responses ranged from 1 (*Never*) to 5 (*All the Time*). One disclosure item was reverse-coded, and all four items were averaged so that higher scores represented greater concealment. Sample items include “I keep secrets from my parents about content I come across on social media” and “I keep secrets from my parents about how often I use social media.” The scale had a Cronbach's alpha of.73.

*Parental media monitoring* was assessed using eight items from the Perceived Parental Media Mediation Scale (42). Although originally developed to assess parental mediation, we use the broader term *parental media monitoring* because the items capture adolescents’ perceptions of parents’ oversight and guidance regarding social media engagement. Items reflected active mediation (e.g., discussing and critically evaluating social media content), restrictive mediation (e.g., limiting time or content), and co-use of social media. Responses ranged from 1 (*Never*) to 5 (*Very Often*). One item was reverse-coded, and items were averaged to create a mean score. Sample items include “The adults that I live with restrict what types of content I can see on social media,” “The adults that I live with talk to me about what I see in social media,” and “I use social media with the adults I live with.” The scale demonstrated good reliability in the current sample (Cronbach's alpha = .84).

Cyberbullying victimization and perpetration were assessed using two dichotomous items adapted from (43) representing verbal forms of online aggression. *Victimization* was assessed with “Someone posted mean or hurtful comments about me online,” whereas *perpetration* was assessed with “I posted mean or hurtful comments about others online.” Participants reported how frequently each experience occurred during the past 30 days from 1 (Never) to 4 (Many Times). Responses were subsequently dichotomized to represent whether each experience occurred (yes) or not experienced (no). These indicators capture involvement in mean or hurtful online interactions rather than a full range of behaviors that may constitute cyberbullying. Although brief, similar single-item dichotomous indicators have been widely used in large epidemiological and school-based surveys to efficiently assess adolescent cyberbullying involvement while minimizing participant burden (44). These items assess involvement in harmful online interactions and are conceptually distinct from the problematic social media use items, which assess compulsive engagement, preoccupation, and interference with functioning rather than aggression or victimization. Because victimization and perpetration were not treated as mutually exclusive, adolescents could be classified as experiencing neither form, victimization only, perpetration only, or both victimization and perpetration for descriptive purposes.

Age and biological sex were included as covariates in the primary models. Age was measured in years, and sex was coded as male or female. Additional sensitivity analyses examined whether the findings were robust to adjustment for race and perceived family financial resources.

### Analysis

Given that the dependent variables were student-reported dichotomies (yes vs. no), logistic generalized linear mixed models (GLMMs) with random intercepts for school were used to account for lack of independence between students nested within schools. This approach was selected because school membership represents a theoretically meaningful source of clustering and conventional logistic regression assumes independence among students within the same school. GLMMs are appropriate for analyzing clustered or nested data (45) and can accommodate categorical outcomes. Intraclass correlation coefficients (ICCs) were calculated from unconditional random-intercept models to quantify the proportion of latent variance in each outcome attributable to between-school differences. For these logistic models, ICCs were calculated using the school-level random-intercept variance and the standard residual variance (π^2^/3).

Separate models were estimated for cyberbullying victimization and perpetration. Primary models included problematic social media use, social media concealment, and parental media monitoring as focal predictors. Age and sex were included as covariates. Models were fit using maximum likelihood via the Laplace approximation. Model coefficients were exponentiated to obtain odds ratios, and 95% confidence intervals were calculated for the odds ratios. Wald *z*-tests were used to evaluate individual fixed-effect estimates. A significance level of.05 was used throughout unless otherwise stated.

Several additional analyses evaluated the robustness of the primary findings. First, exploratory moderation models tested interactions between sex and each focal social media predictor. Second, because parental media monitoring and social media concealment represent conceptually related processes, their correlation and variance inflation factors (VIFs) were examined. Models examining monitoring and concealment separately were then compared descriptively with the primary models containing both predictors.

Finally, sensitivity analyses examined whether the primary findings were robust to additional adjustment for race (White vs. non-White) and perceived family financial resources. Because financial-resource data were unavailable for some participants, these analyses were conducted among the 555 adolescents with complete data. To distinguish changes associated with additional covariate adjustment from those associated with the reduced sample, the primary models were first re-estimated in this same subsample. Race and perceived family financial resources were then added to these models. Perceived family financial resources were treated as an ordered numeric variable ranging from 1 to 4, with higher scores indicating greater perceived financial resources. Likelihood ratio tests were used to evaluate whether jointly adding race and perceived family financial resources improved model fit.

All analyses were conducted in *R*, version 4.4.1 (46) and the ‘*lme4’* package (47) was used to estimate the GLMMs. Fully annotated code and output are available in the Supplementary Materials.

## Results

Descriptive statistics and bivariate correlations are presented in Supplementary Table S2 (supplemental material). Correlations among focal variables were generally small to moderate in magnitude.

Cyberbullying victimization and perpetration showed some overlap in the current sample. Of the 686 adolescents, 91.1% reported neither victimization nor perpetration, 1.5% reported victimization only, 5.4% reported perpetration only, and 2.0% reported both victimization and perpetration. These descriptive patterns indicate meaningful overlap between victimization and perpetration while also supporting examination of the two forms of involvement as distinct outcomes.

In the final models adjusting for age and sex, problematic social media use and social media concealment were significantly associated with both cyberbullying outcomes, whereas parental media monitoring was not significantly associated with either outcome. Age was not significantly associated with victimization or perpetration.

For cyberbullying victimization, males exhibited substantially higher odds of victimization than females, *OR*=5.96, 95% CI [2.15, 16.50]. Problematic social media use was associated with higher odds of victimization, such that a 1-point higher score was associated with more than four times greater odds of victimization, *OR*=4.26, 95% CI [2.06, 8.85]. Social media concealment was also associated, a one-point higher score corresponded to a 77% increase in odds of victimization, *OR*=1.77, 95% CI [1.06, 2.93]. Parental monitoring was not significantly associated with victimization. Panel A of Figure 1 displays these effects, using illustrative values of 1.5 for low, 3.0 for moderate, and 4.5 for high levels for both predictors.

**Figure 1:**
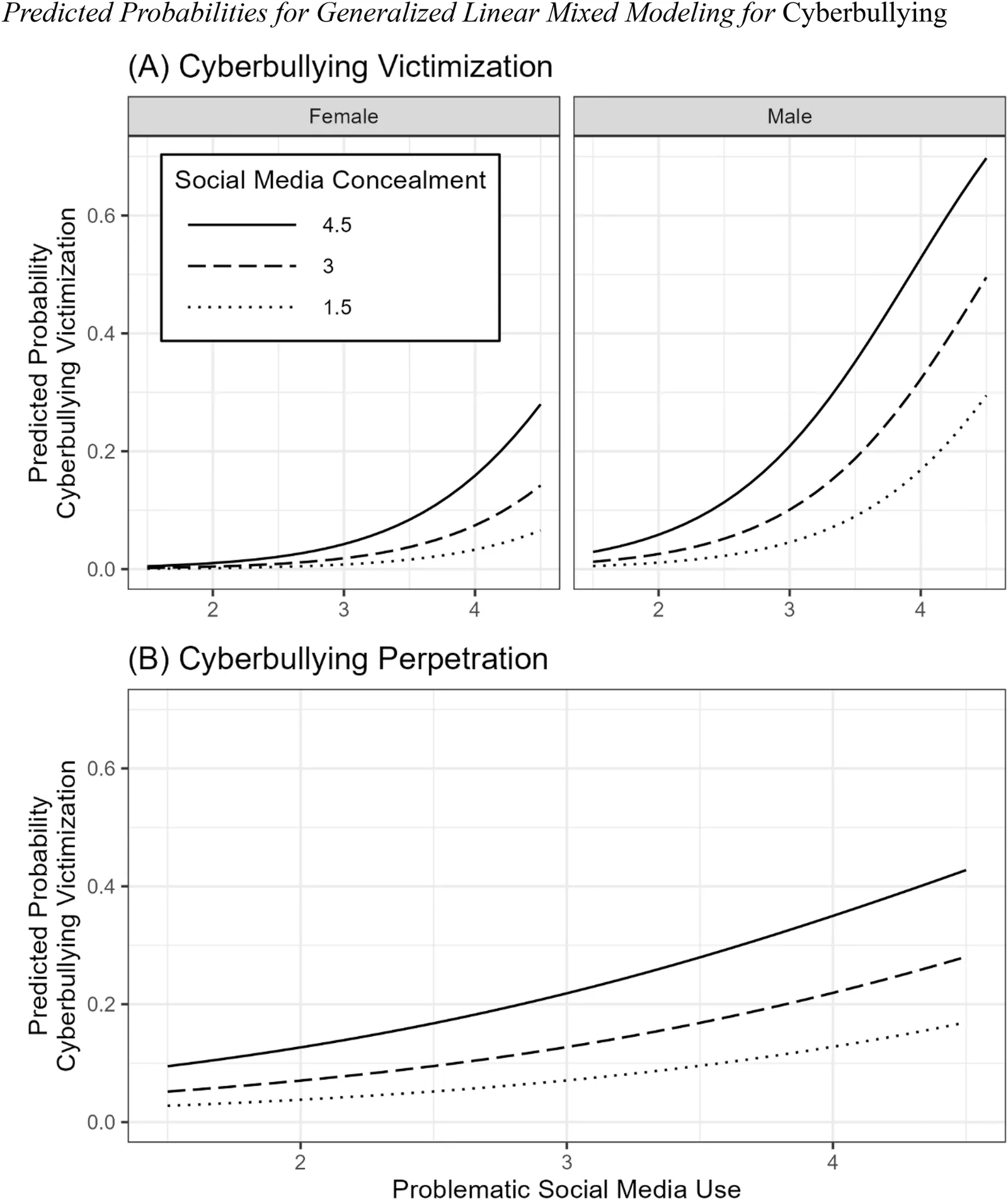
Predicted probabilities for generalized linear mixed modeling for cyberbullying.

For cyberbullying perpetration, problematic social media use was associated with higher odds of perpetration, with a 1-point higher score being associated with nearly double the odds of perpetration, *OR*=1.95, 95% CI [1.24, 3.05]. A 1-point higher score on social media concealment was associated with a 53% increase in the odds of perpetration (OR=1.53, 95% CI [1.07, 2.19]). Parental media monitoring was not significantly associated with perpetration. Complete model coefficients, odds ratios, and 95% confidence intervals are presented in Table 1, and Panel B of Figure 1 displays these effects.

**Table 1:** Parameter estimates for generalized linear mixed modeling for cyberbullying.

Fixed effects	Victimization Model				Perpetration Model			
	*b*		*OR*		*b*		*OR*	
	*Est*	*(SE)*	*Est*	[95% CI]	*Est*	*(SE)*	*Est*	[95% CI]
Intercept	−6.68	(3.03)**	0.00	[0.00, 0.48]	−6.56	(2.28)**	0.00	[0.00, 0.12]
Male vs. Female	**1**.**78**	(0.52)***	**5**.**96**	**[2**.**15, 16**.**50]**	0.52	(0.31)	1.68	[0.91, 3.10]
Age	−0.02	(0.18)	0.98	[0.70, 1.39]	0.10	(0.13)	1.11	[0.86, 1.43]
Problematic Social Media Use	**1**.**45**	(0.37)***	**4**.**26**	**[2**.**06, 8**.**85]**	**0**.**67**	(0.23)**	**1**.**95**	**[1**.**24, 3**.**05]**
Social Media Concealment	**0**.**57**	(0.26)*	**1**.**77**	**[1**.**06, 2**.**93]**	**0**.**42**	(0.18)*	**1**.**53**	**[1**.**07, 2**.**19]**
Parental Monitoring	−0.40	(0.27)	0.67	[0.39, 1.15]	0.01	(0.18)	1.00	[0.70, 1.41]
Random effects	*Var*				*Var*			
School	0.32				0.18			

Est, estimate. Wald procedures were used for parameter significance and confidence interval construction.

* *p* < .05. ** *p* < .01. *** *p* < .001.

Bold values indicate significant effects.

Exploratory moderation analyses indicated that sex did not significantly moderate associations between problematic social media use, parental media monitoring, or social media concealment and either cyberbullying victimization or perpetration (all *p* > .16).

Given the conceptual relationship between parental media monitoring and social media concealment, additional analyses examined their empirical overlap and the robustness of their associations when included simultaneously. Monitoring and concealment were moderately negatively correlated, *r* = −.34, 95% CI [−.40, −.27], *p* < .001. Variance inflation factors provided no evidence of problematic multicollinearity. VIFs for monitoring and concealment were 1.09 and 1.22, respectively, in the victimization model and 1.08 and 1.28 in the perpetration model.

Sensitivity analyses next compared models in which monitoring and concealment were entered separately with the primary models containing both predictors. All models adjusted for age and sex. For victimization, concealment remained significant when modeled without monitoring (*b* = .630, *p* = .011) and when both predictors were included (*b* = .569, *p* = .028). Monitoring was not significant when modeled without concealment (*b* = −.515, *p* = .058) or when both predictors were included (*b* = −.402, *p* = .143). For perpetration, concealment was similarly associated with greater perpetration when modeled without monitoring (*b* = .422, *p* = .018) and when both predictors were included (*b* = .425, *p* = .021). Monitoring was not associated with perpetration when modeled without concealment (*b* = −.082, *p* = .639) or when both predictors were included (*b* = .012, *p* = .948). Thus, although monitoring and concealment were moderately related, the low VIFs and stability of the concealment estimates across models indicated that its associations with victimization and perpetration were robust to the simultaneous inclusion of parental media monitoring.

Sensitivity analyses additionally adjusting for race and perceived family financial resources yielded substantively unchanged results (see Supplementary Table S3 in the supplemental material).

## Discussion

Consistent with our hypotheses, problematic social media use and social media concealment were each associated with greater odds of cyberbullying victimization and perpetration after accounting for school-level clustering. However, contrary to our hypotheses, parental media monitoring was not significantly associated with either outcome. These findings highlight how adolescent social media behaviors are related to cyberbullying involvement. A strength of this study is that data were drawn from 12 schools, allowing the analyses to account for clustering across multiple school contexts. There was also between-school variation in cyberbullying victimization and perpetration, indicating that adolescents’ cyberbullying experiences were not entirely independent of the schools they attended. ICCs from the unconditional models indicated that approximately 10% of the latent variance in victimization and 7% of the latent variance in perpetration was attributed to between-schools. These estimates reflect unexplained between-school heterogeneity rather than effects of specific observed school characteristics. Because school-specific characteristics were not measured in the current study, the factors underlying this variation cannot be explicitly determined. Factors such as school climate, peer norms, or school policies may contribute to this variation and should be directly examined in future research.

Descriptively, some adolescents in the current sample also reported both victimization and perpetration, reinforcing that these cyberbullying roles are not mutually exclusive.

Biological sex was also significantly associated with victimization, with males reporting greater odds of cyberbullying victimization than females. Although some prior studies report higher cybervictimization among girls, findings regarding sex differences vary across samples and forms of cyberbullying (53). The present measure specifically captured mean or hurtful online comments, and additional research is needed to determine whether this finding reflects differences in the forms of cyberaggression experienced or reported by adolescents in this sample. However, sex did not moderate associations between the focal social media variables and either cyberbullying outcome, suggesting that although overall odds of victimization differed by sex, the associations of problematic social media use, parental monitoring, and concealment with cyberbullying did not significantly differ between males and females.

### Social media behaviors

Findings suggest that *how* adolescents use social media is meaningfully associated with adolescents’ cyberbullying involvement. *Problematic social media use* was significantly associated with greater odds of both cyberbullying victimization and perpetration, consistent with prior research linking excessive or addiction-like social media behaviors to heightened vulnerability to online harms (1, 30). Given the strength and consistency of these relationships, digital platforms may be uniquely positioned to help mitigate risk. For example, enhanced digital wellness tools (e.g., screen time tracking, content moderation, behavior-sensitive intervention prompts) have been shown to reduce problematic social media use and improve adolescent digital well-being, which may in turn reduce cyberbullying involvement. While previous research has linked problematic social media use to negative online experiences, the current study demonstrates that problematic social media use is associated with both cyberbullying victimization and perpetration within the same analytic framework, after accounting for school-level clustering. The larger observed association between problematic social media use and victimization (*OR*=4.26), relative to its association with perpetration (*OR*=1.95), may reflect the breadth of this construct, which captures emotional dependence, impaired self-regulation, and interference with relationships and daily functioning, all of which may increase adolescents’ exposure or vulnerability to negative online interactions. However, these differences in magnitude were not formally tested and should therefore be interpreted cautiously. These findings reinforce the importance of prevention efforts that address unhealthy patterns of media engagement rather than focusing solely on screentime.

Similarly, *social media concealment from parents* emerged as a robust predictor of both victimization and perpetration. This finding aligns with previous work indicating that secrecy about media use is associated with increased relational aggression and risk behaviors (36). Importantly, the concealment assessed in this study reflects adolescents’ intentional hiding of social media information from parents, rather than the more general lack of parental knowledge that may naturally occur because parents have limited visibility into adolescents’ online activities. Adolescents who intentionally hide their online behaviors may have fewer opportunities to receive supportive adult guidance or may be engaging in other risky peer contexts. Alternatively, concealment may reflect broader parent-adolescent relationship dynamics, such that adolescents who feel less comfortable communicating openly with parents may be more likely to conceal difficult or risky online experiences. Thus, concealment may function not only as an individual media behavior, but also as an indicator of the quality of parent-adolescent communication surrounding media use. Open family communication about social media may function as a protective proximal process that supports healthier digital habits and potentially reduces cyberbullying involvement (23). Together, these findings support the importance of both individual media behaviors and relational processes in shaping adolescents’ cyberbullying experiences. Because this is one of the first studies to examine social media concealment in relation to cyberbullying, the findings further suggest that secrecy surrounding adolescents’ online activities may represent an overlooked but meaningful indicator of cyberbullying risk that deserves greater attention in future research.

Across both models, problematic social media use and concealment were associated with greater odds of victimization and perpetration. The observed association between problematic social media use and victimization was larger than that for perpetration, although this difference was not formally tested. These findings suggest that problematic engagement and concealment may be relevant to multiple forms of cyberbullying involvement, even if the underlying processes differ across outcomes.

### Parental media monitoring and school settings

Contrary to our hypotheses, parental media monitoring was not independently associated with either cyberbullying victimization or perpetration after accounting for adolescents’ own media behaviors. This contrasts with prior research suggesting that parental oversight can mitigate online risks (6), but should not be interpreted as evidence that parental involvement is unimportant. Parents may have limited direct access to adolescents’ online interactions, particularly when adolescents conceal aspects of their digital activity. One potential explanation is that different parental monitoring strategies have varying effects on cyberbullying risk. Restrictive monitoring (e.g., setting screen time limits or using tracking apps) may inadvertently increase risk by encouraging concealment. In contrast, active monitoring (e.g., open conversations about social media content) and co-use (e.g., parents and adolescents engaging with media together) are more likely to promote trust and reduce secrecy (36). Additionally, the current measure captures adolescents’ perceptions of parental oversight and guidance rather than parents’ actual knowledge of adolescents’ online activities, which may help explain the nonsignificant association. Given that parental monitoring was not directly associated with victimization or perpetration, it is possible that its influence is more nuanced, perhaps operating as a moderator or mediator of the relationship between adolescent media behaviors (e.g., concealment or problematic use) and cyberbullying outcomes. Although monitoring and concealment were examined simultaneously to estimate their unique associations, these processes may also operate together, as greater concealment could limit parents’ ability to effectively monitor adolescents’ online experiences. Consistent with this possibility, the association between parental monitoring and victimization was somewhat attenuated when concealment was included in the model, whereas concealment remained significantly associated with both cyberbullying outcomes across model specifications. Future research should explore how different parental monitoring strategies and adolescents’ concealment operate together in shaping cyberbullying risk to provide more nuanced, evidence-based recommendations for parents.

The school-level random effect indicated between-school variation in cyberbullying involvement; however, the current study did not directly assess school characteristics that might explain these differences. Prior research suggests several potential school-level influences that warrant further investigation. Positive school climates have been linked to lower cyberbullying risk (48), and factors such as peer group norms, digital citizenship, and anti-bullying policies may shape adolescents’ online interactions (49). Moreover, cyberbullying often involves peers or known individuals rather than strangers, mirroring broader patterns seen in in-person bullying literature (26). This challenges the “stranger danger” misconception and highlights the need for school-based programs to holistically address harm that arises within students’ existing social networks. From an ecological perspective, this observed between-school variation highlights the value of future research directly examining which features of school contexts may contribute to differences in cyberbullying involvement (19).

### Limitations

Despite the contributions of the current study, findings should be interpreted within the context of some limitations. First, although there is theoretical reason to believe that social media behaviors may introduce the opportunity for cyberbullying involvement, the cross-sectional design limits our ability to determine directionality between social media use behaviors and cyberbullying. While there is some evidence of bidirectional effects in previous research, longitudinal designs are needed to clarify whether problematic social media use and concealment increase cyberbullying involvement, whether cyberbullying alters subsequent social media behaviors, or whether these processes reinforce one another over time.

Second, cyberbullying victimization and perpetration were measured using single items. Although these items captured experiences of mean or hurtful online comments, cyberbullying encompasses a broader range of behaviors and may include characteristics such as repetition and power imbalance that were not directly assessed in the current study. Future studies should incorporate more comprehensive and multi-item measures to capture the complexity of cyberbullying and improve construct validity. Cyberbullying victimization was also relatively uncommon in the current sample, with only 24 adolescents reporting victimization. The small number of cases may have reduced the precision of estimates in the victimization model, warranting caution when interpreting these results. Research also suggests that cyberbullying and roles frequently overlap, with many adolescents acting as both victims and perpetrators (50). Although the current study descriptively examined overlap between victimization and perpetration, the primary models examined these outcomes separately. Future work could move beyond simple dichotomies to explicitly model victim-perpetrators as a distinct group (51) or consider a composite approach that captures the broader range of cyberbullying involvement (6).

Third, aside from school-level data, all variables in this study were based on adolescent self-reports, which introduces the possibility of social desirability, reporting, and shared method biases. Although the focal measures assessed conceptually distinct behaviors and the problematic social media use items did not directly assess cyberbullying or online aggression, shared method variance may nonetheless have contributed to observed associations. Still, adolescents are often the most reliable source of information about their own social media habits (52), making self-report data appropriate in this context. Future studies would benefit from incorporating multiple informants, such as parent or teacher reports, or observation of social media behavior, to provide a more balanced and comprehensive perspective.

Fourth, although the primary models adjusted for age and sex, other demographic or family characteristics may contribute to cyberbullying involvement. Sensitivity analyses additionally adjusting for race and perceived family financial resources yielded substantively similar findings, but these analyses were limited to participants with complete data. Other potentially relevant factors, such as family structure and academic achievement were not examined. Consequently, residual confounding cannot be ruled out, and future research should examine whether the observed associations remain after accounting for a broader range of individual and family factors.

Finally, the sampling strategy and number of participating schools limit the generalizability and interpretation of the findings. Although all students within participating schools were eligible to participate, district and school participation was nonrandom, and only five of the nine districts approached agreed to participate. Participating districts were also concentrated in more populated areas of the state; consequently, findings may not generalize to adolescents in rural areas or to adolescents outside of the geographic and cultural context represented by this sample. Additionally, although the inclusion of students from 12 schools allowed us to account for the nested structure of the data, the relatively small number of schools may limit the precision of the estimated school-level random-effect variance.

## Future directions and conclusion

Future research should build on these findings by utilizing longitudinal data to clarify developmental pathways and temporal ordering between problematic social media use, concealment, and cyberbullying involvement. Additional work should also examine whether specific parental monitoring strategies function as mediators or moderators of cyberbullying risk and continue to refine the measurement of cyberbullying and adolescents’ online behavior.

This study demonstrates that *how* adolescents engage with social media is associated with greater odds of involvement in cyberbullying. Problematic social media use and concealment emerged as consistent correlates of cyberbullying, whereas parental media monitoring showed a more complex relationship. These findings underscore the need for multi-systemic prevention strategies that move beyond limiting access to social media by promoting healthier patterns of media use, strengthening family communication, supporting safer school and peer environments, and engaging educators, technology developers, and policymakers in creating safer online environments.

## Data Availability

The raw data supporting the conclusions of this article will be made available by the authors, without undue reservation.
