# Supplementary material for: Likes, lies, and oversight: problematic social media use, concealment, parental monitoring, and adolescent cyberbullying
Source: Front Child Adolesc Psychiatry. 2026 Oct 28;5:1933453. doi: 10.3389/frcha.2026.1933453 (PMC13751968; doi:10.3389/frcha.2026.1933453)
Supplement: Supplementary file 1 [file Datasheet1.pdf]

Problematic Social Media Use, Concealment, Parental Monitoring, and Adolescent Cyberbullying

Supplemental Material

September 30, 2026

Contents

1 Preparation **3**

1.1 Packages . . . 3

1.2 Data . . . 4

2 Data Preparation **5**

3 Descriptive Statistics **6**

3.1 Analytic Sample . . . 6

3.1.1 Table S1. Descriptive Statistics by School . . . 7

3.2 Cyberbullying Outcomes and Overlap . . . 9

3.3 Descriptive Statistics by School . . . 10

3.4 Descriptive Statistics for Study Variables . . . 11

4 Correlations **12**

4.1 Table S2. Descriptive Statistics and Bivariate Correlations . . . 12

4.2 Monitoring-Concealment Correlation . . . 15

5 Unconditional Models and School-Level ICCs **16**

6 Primary GLMMs **18**

6.1 Cyberbullying Victimization . . . 18

6.2 Cyberbullying Perpetration . . . 20

7 Odds Ratios and 95% Confidence Intervals **22**

8 Exploratory Sex Moderation Analyses **23**

9 Monitoring and Concealment Sensitivity Analyses **26**

9.1 Victimization . . . 26

9.2 Perpetration . . . 30

10 Sensitivity Analyses with Additional Covariates **33**
10.1 Victimization . . . 34
10.2 Perpetration . . . 37

11 Session Information **43**

List of Tables

1 Descriptive Statistics by School . . . 8
2 Sample Size and Age Characteristics by School . . . 10
3 Descriptive Statistics for Study Variables . . . 11
4 Descriptive Statistics and Bivariate Correlations . . . 13

List of Figures

1 Preparation

1.1 Packages

```r
library(tidyverse)
library(furniture)
library(lme4)
library(performance)
library(car)
```

1.2 Data

```r
dfl <- read_csv("CHECKED_prepped_data.csv") %>%
  dplyr::group_by(pid) %>%
  dplyr::mutate(nid = row_number()) %>%
  dplyr::ungroup() %>%
  dplyr::select(pid, nid, everything())

# "I'm not sure" / non-substantive financial-resource response is missing.
dfl$money[dfl$money == 999] <- NA
```

2 Data Preparation

```
df_use <- dfl %>%
  dplyr::mutate(
    agg = ifelse(bully_1 > 2, 1, 0),
    victim = ifelse(bully_2 > 2, 1, 0),
    mean_bully = rowmeans(bully_1, bully_2),
    risk = ifelse(mean_bully > 2, 1, 0),
    id = factor(paste(pid, school, sep = "_")),
    school = factor(school),
    grade = factor(grade),
    race = factor(race),
    money = factor(money),
    agg = factor(agg, levels = c(0, 1), labels = c("No", "Yes")),
    victim = factor(victim, levels = c(0, 1), labels = c("No", "Yes")),
    risk = factor(risk, levels = c(0, 1), labels = c("No", "Yes")),
    sex = factor(sex, levels = c(1, 2), labels = c("Male", "Female")),
    white = case_when(
      race == "1" ~ 1,
      TRUE ~ 0
    ) %>% factor(levels = c(0, 1), labels = c("No", "Yes")),
    mean_conceal = rowmeans.n(
      hide_1, hide_2, hide_3, 6 - hide_4,
      n = 1
    ),
    mean_monitor = rowmeans.n(
      monitor_1, monitor_2, monitor_3, monitor_4,
      monitor_5, monitor_6, monitor_7, 6 - monitor_8,
      n = 1
    ),
    mean_probsm = rowmeans.n(
      probsm_1, probsm_2, probsm_3, probsm_4,
      probsm_5, probsm_6, probsm_7,
      n = 1
    ),
    nmiss = rowsums(
      is.na(hide_1), is.na(hide_2), is.na(hide_3), is.na(hide_4),
      is.na(monitor_1), is.na(monitor_2), is.na(monitor_3), is.na(monitor_4),
      is.na(monitor_5), is.na(monitor_6), is.na(monitor_7), is.na(monitor_8),
      is.na(bully_1), is.na(bully_2),
      is.na(probsm_1), is.na(probsm_2), is.na(probsm_3), is.na(probsm_4),
      is.na(probsm_5), is.na(probsm_6), is.na(probsm_7),
      is.na(risk), is.na(agg), is.na(victim)
    )
  ) %>%
  dplyr::filter(nmiss < 24) %>%
  dplyr::filter(!is.na(risk)) %>%
  dplyr::select(
    id, pid, schoolF, school, white, grade, money, age, sex, race,
    agg, victim, risk, nmiss, starts_with("mean"),
    probsm_1:probsm_7, hide_1:hide_4, monitor_1:monitor_8,
    bully_1:bully_2, everything(), -monitor_9
  )
```

3 Descriptive Statistics

3.1 Analytic Sample

```r
nrow(df_use)
```

```
## [1] 686
```

```r
table(df_use$sex, useNA = "ifany")
```

```
##
##   Male Female
##    280    406
```

```r
summary(df_use$age)
```

```
##    Min. 1st Qu.  Median    Mean 3rd Qu.    Max.
##   12.00   14.00   15.00   15.21   16.00   18.00
```

```r
table(df_use$white, useNA = "ifany")
```

```
##
##  No Yes
## 146 540
```

```r
table(df_use$money, useNA = "ifany")
```

```
##
##    1    2    3    4 <NA>
##   11   75  234  235  131
```

3.1.1 Table S1. Descriptive Statistics by School

```
school_lookup <- df_use %>%
  dplyr::distinct(school) %>%
  dplyr::arrange(school) %>%
  dplyr::mutate(school_number = dplyr::row_number())

table_s1 <- df_use %>%
  dplyr::mutate(
    school_number = paste0(
      "School ",
      as.integer(factor(school))
    )
  ) %>%
  dplyr::group_by(school_number) %>%
  dplyr::summarise(
    N = dplyr::n(),
    `Age M` = mean(age, na.rm = TRUE),
    `Age SD` = sd(age, na.rm = TRUE),
    `PMU M` = mean(mean_probsm, na.rm = TRUE),
    `PMU SD` = sd(mean_probsm, na.rm = TRUE),
    `Concealment M` = mean(mean_conceal, na.rm = TRUE),
    `Concealment SD` = sd(mean_conceal, na.rm = TRUE),
    `Monitoring M` = mean(mean_monitor, na.rm = TRUE),
    `Monitoring SD` = sd(mean_monitor, na.rm = TRUE),
    `% Victimization` = mean(victim == "Yes", na.rm = TRUE) * 100,
    `% Perpetration` = mean(agg == "Yes", na.rm = TRUE) * 100,
    .groups = "drop"
  ) %>%
  dplyr::mutate(
    Age = sprintf("%.2f (%.2f)", `Age M`, `Age SD`),
    PMU = sprintf("%.2f (%.2f)", `PMU M`, `PMU SD`),
    Concealment = sprintf(
      "%.2f (%.2f)",
      `Concealment M`,
      `Concealment SD`
    ),
    Monitoring = sprintf(
      "%.2f (%.2f)",
      `Monitoring M`,
      `Monitoring SD`
    ),
    `% Victimization` = sprintf("%.1f", `% Victimization`),
    `% Perpetration` = sprintf("%.1f", `% Perpetration`)
  ) %>%
  dplyr::select(
    School = school_number,
    N,
    Age,
    PMU,
    Concealment,
    Monitoring,
    `% Victimization`,
```

Table 1: Descriptive Statistics by School

School	N	Age M(SD)	PMU M(SD)	Conceal M(SD)	Monitor M(SD)	Victim (%)	Perpetrate(%)
School 1	137	13.91 (1.04)	2.35 (0.71)	1.86 (0.70)	3.53 (0.87)	0.0	5.1
School 10	32	17.22 (0.71)	2.54 (0.71)	2.01 (0.57)	3.05 (0.88)	0.0	9.4
School 11	59	14.80 (0.98)	2.57 (0.93)	2.35 (1.01)	2.90 (0.89)	6.8	22.0
School 12	41	14.83 (1.05)	2.54 (0.54)	2.01 (0.77)	3.27 (0.78)	7.3	4.9
School 2	149	16.67 (0.98)	2.47 (0.68)	1.96 (0.74)	3.47 (0.83)	4.0	2.7
School 3	47	16.79 (1.06)	2.68 (0.67)	2.32 (0.84)	2.44 (0.82)	4.3	6.4
School 4	25	14.88 (1.88)	2.68 (0.79)	1.88 (0.78)	3.41 (0.77)	0.0	4.0
School 5	48	14.54 (0.92)	2.53 (0.65)	2.05 (0.86)	3.28 (0.88)	8.3	8.3
School 6	75	14.45 (0.92)	2.47 (0.65)	1.87 (0.79)	3.41 (0.94)	4.0	8.0
School 7	32	14.38 (0.98)	2.48 (0.65)	1.76 (0.60)	3.57 (0.74)	0.0	9.4
School 8	15	15.73 (1.58)	2.41 (0.70)	2.17 (0.95)	3.27 (0.90)	0.0	13.3
School 9	26	14.46 (0.86)	2.71 (0.72)	2.13 (0.73)	3.39 (0.95)	7.7	11.5

```
    `% Perpetration`
  )

knitr::kable(
  table_s1,
  format = "latex",
  booktabs = TRUE,
  caption = "Descriptive Statistics by School",
  col.names = c(
    "School",
    "N",
    "Age M(SD)",
    "PMU M(SD)",
    "Conceal M(SD)",
    "Monitor M(SD)",
    "Victim (%)",
    "Perpetrate(%)"
  ),
  align = c("l", "c", "c", "c", "c", "c", "c", "c")
)
```

3.2 Cyberbullying Outcomes and Overlap

```
table(df_use$victim, useNA = "ifany")
```

```
##
##  No Yes
## 662  24
```

```
table(df_use$agg, useNA = "ifany")
```

```
##
##  No Yes
## 635  51
```

```
table(df_use$victim, df_use$agg)
```

```
##
##         No Yes
##   No   625  37
##   Yes   10  14
```

```
prop.table(table(df_use$victim, df_use$agg))
```

```
##
##                 No        Yes
##   No  0.91107872 0.05393586
##   Yes 0.01457726 0.02040816
```

Table 2: Sample Size and Age Characteristics by School

School	N	Minimum Age	Maximum Age	Mean Age
School 1	137	12	18	13.91
School 2	149	15	18	16.67
School 3	47	14	18	16.79
School 4	25	12	18	14.88
School 5	48	13	16	14.54
School 6	75	13	16	14.45
School 7	32	13	16	14.38
School 8	15	13	18	15.73
School 9	26	13	16	14.46
School 10	32	16	18	17.22
School 11	59	13	16	14.80
School 12	41	13	16	14.83

3.3 Descriptive Statistics by School

```
school_descriptives <- df_use %>%
  dplyr::mutate(
    school_number = as.integer(factor(school))
  ) %>%
  dplyr::group_by(school_number) %>%
  dplyr::summarise(
    N = dplyr::n(),
    `Minimum Age` = min(age, na.rm = TRUE),
    `Maximum Age` = max(age, na.rm = TRUE),
    `Mean Age` = mean(age, na.rm = TRUE),
    .groups = "drop"
  ) %>%
  dplyr::mutate(
    School = paste0("School ", school_number),
    `Mean Age` = sprintf("%.2f", `Mean Age`)
  ) %>%
  dplyr::select(
    School,
    N,
    `Minimum Age`,
    `Maximum Age`,
    `Mean Age`
  )

knitr::kable(
  school_descriptives,
  format = "latex",
  booktabs = TRUE,
  caption = "Sample Size and Age Characteristics by School",
  align = c("l", "c", "c", "c", "c")
)
```

3.4 Descriptive Statistics for Study Variables

```
df_use %>%
  dplyr::select(
    age, sex, mean_probsm, mean_conceal, mean_monitor,
    victim, agg
  ) %>%
  furniture::table1(
    digits = 3,
    total = TRUE,
    na.rm = TRUE,
    output = "markdown",
    caption = "Descriptive Statistics for Study Variables"
  )
```

Table 3: Descriptive Statistics for Study Variables

	Mean/Count (SD/%)
	n = 686
age	
	15.213 (1.550)
sex	
Male	280 (40.8%)
Female	406 (59.2%)
mean_probsm	
	2.497 (0.706)
mean_conceal	
	1.998 (0.788)
mean_monitor	
	3.306 (0.903)
victim	
No	662 (96.5%)
Yes	24 (3.5%)
agg	
No	635 (92.6%)
Yes	51 (7.4%)

4 Correlations

4.1 Table S2. Descriptive Statistics and Bivariate Correlations

```
# Prepare variables for correlation table
table_s2_data <- df_use %>%
  dplyr::transmute(
    age = age,
    sex = ifelse(sex == "Female", 1, 0),
    pmu = mean_probsm,
    concealment = mean_conceal,
    monitoring = mean_monitor,
    victimization = ifelse(victim == "Yes", 1, 0),
    perpetration = ifelse(agg == "Yes", 1, 0)
  )

# Variable labels
var_labels <- c(
  "1. Age",
  "2. Sex",
  "3. Problematic social media use",
  "4. Social media concealment",
  "5. Parental media monitoring",
  "6. Cyberbullying victimization",
  "7. Cyberbullying perpetration"
)

# Descriptive statistics
means <- sapply(table_s2_data, mean, na.rm = TRUE)
sds   <- sapply(table_s2_data, sd, na.rm = TRUE)

# Correlations and p-values
corr_results <- psych::corr.test(
  table_s2_data,
  use = "pairwise",
  method = "pearson",
  adjust = "none"
)

r_mat <- corr_results$r
p_mat <- corr_results$p

# Function to format correlations with significance stars
format_r <- function(r, p) {
  stars <- dplyr::case_when(
    p < .001 ~ "***",
    p < .01  ~ "**",
    p < .05  ~ "*",
    TRUE     ~ ""
  )

  paste0(sprintf("%.2f", r), stars)
}
```

Table 4: Descriptive Statistics and Bivariate Correlations

Variable	M	SD	1	2	3	4	5	6	7
1. Age	15.21	1.55							
2. Sex	0.59	0.49	-0.05						
3. Problematic social media use	2.50	0.71	0.07	0.13***					
4. Social media concealment	2.00	0.79	0.10**	-0.08*	0.36***				
5. Parental media monitoring	3.31	0.90	-0.14***	0.10**	-0.08*	-0.34***			
6. Cyberbullying victimization	0.03	0.18	0.03	-0.12**	0.20***	0.21***	-0.08*		
7. Cyberbullying perpetration	0.07	0.26	0.01	-0.05	0.17***	0.18***	-0.05	0.37***	

```r
# Create lower-triangle correlation table
cor_display <- matrix(
  "",
  nrow = ncol(table_s2_data),
  ncol = ncol(table_s2_data)
)

for (i in 2:nrow(cor_display)) {
  for (j in 1:(i - 1)) {
    cor_display[i, j] <- format_r(
      r_mat[i, j],
      p_mat[i, j]
    )
  }
}

# Assemble final table
table_s2 <- data.frame(
  Variable = var_labels,
  M = sprintf("%.2f", means),
  SD = sprintf("%.2f", sds),
  cor_display,
  check.names = FALSE
)

names(table_s2)[4:10] <- as.character(1:7)

# Print Table S2
knitr::kable(
  table_s2,
  format = "latex",
  booktabs = TRUE,
  caption = "Descriptive Statistics and Bivariate Correlations",
  align = c("l", rep("c", 9)),
  escape = FALSE
)
```

Note. N = 686. Sex was coded 0 = male and 1 = female. Cyberbullying victimization and perpetration were coded 0 = no and 1 = yes. Correlations involving a dichotomous and continuous variable are point-biserial correlations; the correlation between the two dichotomous cyberbullying variables is a phi coefficient. $p <$

.05. $\mathbf{p} < .01$. $\boldsymbol{p} < .001$.

4.2 Monitoring-Concealment Correlation

```r
cor.test(df_use$mean_conceal, df_use$mean_monitor)
```

```
##
##  Pearson's product-moment correlation
##
## data:  df_use$mean_conceal and df_use$mean_monitor
## t = -9.4679, df = 684, p-value < 2.2e-16
## alternative hypothesis: true correlation is not equal to 0
## 95 percent confidence interval:
##  -0.4049343 -0.2724841
## sample estimates:
##        cor
## -0.3403967
```

5 Unconditional Models and School-Level ICCs

```
victim_null <- lme4::glmer(
  victim ~ 1 + (1 | school),
  data = df_use,
  family = binomial(link = "logit")
)

agg_null <- lme4::glmer(
  agg ~ 1 + (1 | school),
  data = df_use,
  family = binomial(link = "logit")
)

summary(victim_null)
```

```
## Generalized linear mixed model fit by maximum likelihood (Laplace
##   Approximation) [glmerMod]
##  Family: binomial  ( logit )
## Formula: victim ~ 1 + (1 | school)
##    Data: df_use
##
##       AIC       BIC    logLik -2*log(L)  df.resid
##     210.4     219.5    -103.2     206.4       684
##
## Scaled residuals:
##     Min      1Q  Median      3Q     Max
## -0.2343 -0.1962 -0.1917 -0.1580  5.2196
##
## Random effects:
##  Groups Name        Variance Std.Dev.
##  school (Intercept) 0.3457   0.588
## Number of obs: 686, groups:  school, 12
##
## Fixed effects:
##             Estimate Std. Error z value Pr(>|z|)
## (Intercept)  -3.4210     0.3191  -10.72   <2e-16 ***
## ---
## Signif. codes:  0 '***' 0.001 '**' 0.01 '*' 0.05 '.' 0.1 ' ' 1
```

```
summary(agg_null)
```

```
## Generalized linear mixed model fit by maximum likelihood (Laplace
##   Approximation) [glmerMod]
##  Family: binomial  ( logit )
## Formula: agg ~ 1 + (1 | school)
##    Data: df_use
##
##       AIC       BIC    logLik -2*log(L)  df.resid
##     361.2     370.2    -178.6     357.2       684
##
```

```
## Scaled residuals:
##     Min      1Q  Median      3Q     Max
## -0.4367 -0.2920 -0.2626 -0.2120  4.7163
##
## Random effects:
##  Groups Name        Variance Std.Dev.
##  school (Intercept) 0.2425   0.4924
## Number of obs: 686, groups:  school, 12
##
## Fixed effects:
##             Estimate Std. Error z value Pr(>|z|)
## (Intercept)  -2.5176     0.2142  -11.75   <2e-16 ***
## ---
## Signif. codes:  0 '***' 0.001 '**' 0.01 '*' 0.05 '.' 0.1 ' ' 1
```

```r
performance::icc(victim_null)
```

```
## # Intraclass Correlation Coefficient
##
##     Adjusted ICC: 0.095
##   Unadjusted ICC: 0.095
```

```r
performance::icc(agg_null)
```

```
## # Intraclass Correlation Coefficient
##
##     Adjusted ICC: 0.069
##   Unadjusted ICC: 0.069
```

6 Primary GLMMs

6.1 Cyberbullying Victimization

```r
victim_primary_start <- lme4::glmer(
  victim ~ sex + age + mean_probsm + mean_monitor + mean_conceal +
    (1 | school),
  data = df_use,
  family = binomial(link = "logit"),
  control = glmerControl(
    optimizer = "Nelder_Mead",
    optCtrl = list(maxfun = 100000)
  )
)

# Optimizer check; final estimates use the converged Nelder-Mead solution.
victim_primary_allfit <- lme4::allFit(victim_primary_start)
```

```
## bobyqa : [OK]
## Nelder_Mead : [OK]
## nlminbwrap : [OK]
## optimx.L-BFGS-B :

## [OK]
## nloptwrap.NLOPT_LN_NELDERMEAD : [OK]
## nloptwrap.NLOPT_LN_BOBYQA :

## [OK]
```

```r
victim_primary <- victim_primary_allfit$Nelder_Mead

summary(victim_primary)
```

```
## Generalized linear mixed model fit by maximum likelihood (Laplace
##   Approximation) [glmerMod]
##  Family: binomial  ( logit )
## Formula: victim ~ sex + age + mean_probsm + mean_monitor + mean_conceal +
##     (1 | school)
##    Data: df_use
## Control: ctrl
##
##       AIC       BIC    logLik -2*log(L)  df.resid
##     170.4     202.1     -78.2     156.4       679
##
## Scaled residuals:
##     Min      1Q  Median      3Q     Max
## -1.1137 -0.1697 -0.0981 -0.0550  8.2603
##
## Random effects:
##  Groups Name        Variance Std.Dev.
##  school (Intercept) 0.3347   0.5786
```

```
## Number of obs: 686, groups:  school, 12
##
## Fixed effects:
##              Estimate Std. Error z value Pr(>|z|)
## (Intercept)  -6.67840    3.03293  -2.202 0.027668 *
## sexFemale    -1.78449    0.51985  -3.433 0.000598 ***
## age          -0.01609    0.17592  -0.091 0.927120
## mean_probsm   1.45030    0.37242   3.894 9.85e-05 ***
## mean_monitor -0.40224    0.27496  -1.463 0.143491
## mean_conceal  0.56888    0.25851   2.201 0.027761 *
## ---
## Signif. codes:  0 '***' 0.001 '**' 0.01 '*' 0.05 '.' 0.1 ' ' 1
##
## Correlation of Fixed Effects:
##             (Intr) sexFml age    mn_prb mn_mnt
## sexFemale   -0.058
## age         -0.907  0.089
## mean_probsm -0.239 -0.240  0.021
## mean_monitr -0.304  0.088  0.117 -0.255
## mean_concel -0.053 -0.006 -0.069 -0.403  0.150
```

```
lme4::isSingular(victim_primary)
```

```
## [1] FALSE
```

```
VarCorr(victim_primary)
```

```
##  Groups Name        Std.Dev.
##  school (Intercept) 0.57856
```

```
car::vif(victim_primary)
```

```
##          sex          age  mean_probsm mean_monitor mean_conceal
##     1.084934     1.029796     1.339729     1.091777     1.219254
```

6.2 Cyberbullying Perpetration

```
agg_primary <- lme4::glmer(
  agg ~ sex + age + mean_probsm + mean_monitor + mean_conceal +
    (1 | school),
  data = df_use,
  family = binomial(link = "logit"),
  control = glmerControl(
    optimizer = "bobyqa",
    optCtrl = list(maxfun = 100000)
  )
)

summary(agg_primary)
```

```
## Generalized linear mixed model fit by maximum likelihood (Laplace
##   Approximation) [glmerMod]
##  Family: binomial  ( logit )
## Formula: agg ~ sex + age + mean_probsm + mean_monitor + mean_conceal +
##     (1 | school)
##    Data: df_use
## Control: glmerControl(optimizer = "bobyqa", optCtrl = list(maxfun = 1e+05))
##
##       AIC       BIC    logLik -2*log(L)  df.resid
##     344.3     376.0    -165.1     330.3       679
##
## Scaled residuals:
##     Min      1Q  Median      3Q     Max
## -1.0823 -0.2905 -0.2228 -0.1661 10.2111
##
## Random effects:
##  Groups Name        Variance Std.Dev.
##  school (Intercept) 0.2375   0.4873
## Number of obs: 686, groups:  school, 12
##
## Fixed effects:
##              Estimate Std. Error z value Pr(>|z|)
## (Intercept)   -6.5596     2.2788  -2.879  0.00400 **
## sexFemale     -0.5207     0.3110  -1.674  0.09408 .
## age            0.1029     0.1308   0.787  0.43132
## mean_probsm    0.6660     0.2292   2.906  0.00366 **
## mean_monitor   0.0118     0.1793   0.066  0.94754
## mean_conceal   0.4248     0.1838   2.311  0.02083 *
## ---
## Signif. codes:  0 '***' 0.001 '**' 0.01 '*' 0.05 '.' 0.1 ' ' 1
##
## Correlation of Fixed Effects:
##             (Intr) sexFml age    mn_prb mn_mnt
## sexFemale   -0.066
## age         -0.925  0.048
## mean_probsm -0.186 -0.200  0.026
## mean_monitr -0.386 -0.023  0.142 -0.106
## mean_concel -0.110  0.088 -0.026 -0.425  0.230
```

```r
lme4::isSingular(agg_primary)
```

```
## [1] FALSE
```

```r
VarCorr(agg_primary)
```

```
##  Groups Name        Std.Dev.
##  school (Intercept) 0.48734
```

```r
car::vif(agg_primary)
```

```
##          sex          age  mean_probsm mean_monitor mean_conceal
##     1.048163     1.028683     1.262546     1.084247     1.276894
```

7 Odds Ratios and 95% Confidence Intervals

```
# Victimization
exp(cbind(
  OR = fixef(victim_primary),
  confint(victim_primary, parm = "beta_", method = "Wald")
))
```

```
##                       OR        2.5 %    97.5 %
## (Intercept)  0.001257793 3.295884e-06 0.4800058
## sexFemale    0.167882590 6.060511e-02 0.4650526
## age          0.984037557 6.970585e-01 1.3891660
## mean_probsm  4.264375401 2.055168e+00 8.8483750
## mean_monitor 0.668823053 3.901833e-01 1.1464464
## mean_conceal 1.766292660 1.064196e+00 2.9315937
```

```
# Perpetration
exp(cbind(
  OR = fixef(agg_primary),
  confint(agg_primary, parm = "beta_", method = "Wald")
))
```

```
##                       OR       2.5 %    97.5 %
## (Intercept)  0.001416409 0.000016272 0.1232924
## sexFemale    0.594083810 0.322923227 1.0929396
## age          1.108399564 0.857775907 1.4322501
## mean_probsm  1.946353271 1.242102033 3.0499033
## mean_monitor 1.011869471 0.711979245 1.4380754
## mean_conceal 1.529248536 1.066657498 2.1924574
```

8 Exploratory Sex Moderation Analyses

The following models test whether sex moderates associations between each focal social media predictor and cyberbullying. Age is retained as a covariate.

```r
victim_sex_interactions <- lme4::glmer(
  victim ~ age + sex * mean_probsm + sex * mean_monitor + sex * mean_conceal +
    (1 | school),
  data = df_use,
  family = binomial(link = "logit"),
  control = glmerControl(
    optimizer = "bobyqa",
    optCtrl = list(maxfun = 200000)
  )
)

agg_sex_interactions <- lme4::glmer(
  agg ~ age + sex * mean_probsm + sex * mean_monitor + sex * mean_conceal +
    (1 | school),
  data = df_use,
  family = binomial(link = "logit"),
  control = glmerControl(
    optimizer = "bobyqa",
    optCtrl = list(maxfun = 200000)
  )
)

summary(victim_sex_interactions)
```

```
## Generalized linear mixed model fit by maximum likelihood (Laplace
##   Approximation) [glmerMod]
##  Family: binomial  ( logit )
## Formula: victim ~ age + sex * mean_probsm + sex * mean_monitor + sex *
##     mean_conceal + (1 | school)
##    Data: df_use
## Control: glmerControl(optimizer = "bobyqa", optCtrl = list(maxfun = 2e+05))
##
##       AIC       BIC    logLik -2*log(L)  df.resid
##     173.7     219.0     -76.8     153.7       676
##
## Scaled residuals:
##     Min      1Q  Median      3Q     Max
## -0.9954 -0.1689 -0.0929 -0.0399  8.9829
##
## Random effects:
##  Groups Name        Variance Std.Dev.
##  school (Intercept) 0.4368   0.6609
## Number of obs: 686, groups:  school, 12
##
## Fixed effects:
##                         Estimate Std. Error z value Pr(>|z|)
## (Intercept)             -7.09963    3.23378  -2.195 0.028130 *
## age                     -0.02480    0.18329  -0.135 0.892364
```

```
## sexFemale              -0.20159    2.82745  -0.071 0.943162
## mean_probsm             1.42894    0.43414   3.291 0.000997 ***
## mean_monitor           -0.11393    0.32977  -0.345 0.729724
## mean_conceal            0.45558    0.32022   1.423 0.154829
## sexFemale:mean_probsm   0.01987    0.88858   0.022 0.982158
## sexFemale:mean_monitor -0.85635    0.60844  -1.407 0.159293
## sexFemale:mean_conceal  0.27803    0.58378   0.476 0.633890
## ---
## Signif. codes:  0 '***' 0.001 '**' 0.01 '*' 0.05 '.' 0.1 ' ' 1
##
## Correlation of Fixed Effects:
##             (Intr) age    sexFml mn_prb mn_mnt mn_cnc sxFml:mn_p sxFml:mn_m
## age         -0.871
## sexFemale   -0.218 -0.051
## mean_probsm -0.327  0.083  0.276
## mean_monitr -0.309  0.047  0.311 -0.192
## mean_concel -0.001 -0.159  0.168 -0.371  0.116
## sxFml:mn_pr  0.189 -0.091 -0.584 -0.472  0.084  0.187
## sxFml:mn_mn  0.068  0.110 -0.420  0.089 -0.529 -0.077 -0.267
## sxFml:mn_cn -0.057  0.167 -0.207  0.199 -0.070 -0.560 -0.476      0.181
```

```r
summary(agg_sex_interactions)
```

```
## Generalized linear mixed model fit by maximum likelihood (Laplace
##   Approximation) [glmerMod]
##  Family: binomial  ( logit )
## Formula:
## agg ~ age + sex * mean_probsm + sex * mean_monitor + sex * mean_conceal +
##     (1 | school)
##    Data: df_use
## Control: glmerControl(optimizer = "bobyqa", optCtrl = list(maxfun = 2e+05))
##
##       AIC       BIC    logLik -2*log(L)  df.resid
##     348.9     394.3    -164.5     328.9       676
##
## Scaled residuals:
##     Min      1Q  Median      3Q     Max
## -1.3373 -0.2881 -0.2189 -0.1672  9.1088
##
## Random effects:
##  Groups Name        Variance Std.Dev.
##  school (Intercept) 0.2713   0.5208
## Number of obs: 686, groups:  school, 12
##
## Fixed effects:
##                        Estimate Std. Error z value Pr(>|z|)
## (Intercept)            -7.14978    2.42092  -2.953  0.00314 **
## age                     0.09081    0.13249   0.685  0.49309
## sexFemale               1.05914    1.75359   0.604  0.54586
## mean_probsm             0.69857    0.31989   2.184  0.02898 *
## mean_monitor            0.08732    0.26329   0.332  0.74016
## mean_conceal            0.61035    0.25886   2.358  0.01838 *
## sexFemale:mean_probsm  -0.05560    0.46297  -0.120  0.90440
## sexFemale:mean_monitor -0.17434    0.35560  -0.490  0.62394
```

```
## sexFemale:mean_conceal -0.37214    0.37719  -0.987  0.32383
## ---
## Signif. codes:  0 '***' 0.001 '**' 0.01 '*' 0.05 '.' 0.1 ' ' 1
##
## Correlation of Fixed Effects:
##             (Intr) age    sexFml mn_prb mn_mnt mn_cnc sxFml:mn_p sxFml:mn_m
## age         -0.866
## sexFemale   -0.324 -0.023
## mean_probsm -0.269  0.076  0.280
## mean_monitr -0.364  0.046  0.453 -0.182
## mean_concel -0.126 -0.089  0.290 -0.352  0.188
## sxFml:mn_pr  0.218 -0.088 -0.468 -0.696  0.121  0.247
## sxFml:mn_mn  0.181  0.068 -0.683  0.142 -0.729 -0.154 -0.113
## sxFml:mn_cn  0.060  0.107 -0.365  0.240 -0.149 -0.704 -0.404      0.254
```

9 Monitoring and Concealment Sensitivity Analyses

These models evaluate whether the associations for parental media monitoring and social media concealment are robust when the two predictors are entered separately versus simultaneously. All models adjust for age, sex, and problematic social media use.

9.1 Victimization

```
victim_conceal_start <- lme4::glmer(
  victim ~ sex + age + mean_probsm + mean_conceal + (1 | school),
  data = df_use,
  family = binomial(link = "logit"),
  control = glmerControl(
    optimizer = "Nelder_Mead",
    optCtrl = list(maxfun = 100000)
  )
)

# Optimizer check used because of the rare victimization outcome.
victim_conceal_allfit <- lme4::allFit(victim_conceal_start)
```

```
## bobyqa : [OK]
## Nelder_Mead :

## [OK]
## nlminbwrap :

## [OK]
## optimx.L-BFGS-B :

## [OK]
## nloptwrap.NLOPT_LN_NELDERMEAD :

## [OK]
## nloptwrap.NLOPT_LN_BOBYQA :

## [OK]
```

```
victim_conceal <- victim_conceal_allfit$bobyqa

victim_monitor <- lme4::glmer(
  victim ~ sex + age + mean_probsm + mean_monitor + (1 | school),
  data = df_use,
  family = binomial(link = "logit"),
  control = glmerControl(
    optimizer = "Nelder_Mead",
    optCtrl = list(maxfun = 100000)
  )
)

summary(victim_conceal)
```

```
## Generalized linear mixed model fit by maximum likelihood (Laplace
##   Approximation) [glmerMod]
##  Family: binomial  ( logit )
## Formula: victim ~ sex + age + mean_probsm + mean_conceal + (1 | school)
##    Data: df_use
## Control: ctrl
##
##      AIC       BIC    logLik -2*log(L)  df.resid
##    170.6     197.8     -79.3     158.6       680
##
## Scaled residuals:
##     Min      1Q  Median      3Q     Max
## -1.1883 -0.1711 -0.1038 -0.0628  8.8467
##
## Random effects:
##  Groups Name        Variance Std.Dev.
##  school (Intercept) 0.2787   0.5279
## Number of obs: 686, groups:  school, 12
##
## Fixed effects:
##              Estimate Std. Error z value Pr(>|z|)
## (Intercept)  -8.15934    2.86580  -2.847 0.004411 **
## sexFemale    -1.73431    0.51174  -3.389 0.000701 ***
## age           0.01395    0.17252   0.081 0.935561
## mean_probsm   1.33582    0.35164   3.799 0.000145 ***
## mean_conceal  0.63025    0.24749   2.547 0.010879 *
## ---
## Signif. codes:  0 '***' 0.001 '**' 0.01 '*' 0.05 '.' 0.1 ' ' 1
##
## Correlation of Fixed Effects:
##             (Intr) sexFml age    mn_prb
## sexFemale   -0.058
## age         -0.927  0.101
## mean_probsm -0.349 -0.231  0.070
## mean_concel -0.002  0.011 -0.085 -0.393
```

```
summary(victim_monitor)
```

```
## Generalized linear mixed model fit by maximum likelihood (Laplace
##   Approximation) [glmerMod]
##  Family: binomial  ( logit )
## Formula: victim ~ sex + age + mean_probsm + mean_monitor + (1 | school)
##    Data: df_use
## Control:
## glmerControl(optimizer = "Nelder_Mead", optCtrl = list(maxfun = 1e+05))
##
##      AIC       BIC    logLik -2*log(L)  df.resid
##    173.1     200.3     -80.5     161.1       680
##
## Scaled residuals:
##     Min      1Q  Median      3Q     Max
## -0.8521 -0.1752 -0.0989 -0.0534  9.5568
##
## Random effects:
```

```
##  Groups Name        Variance Std.Dev.
##  school (Intercept) 0.4174   0.646
## Number of obs: 686, groups:  school, 12
##
## Fixed effects:
##               Estimate Std. Error z value Pr(>|z|)
## (Intercept)  -6.404422   3.021015  -2.120 0.034010 *
## sexFemale    -1.821686   0.505507  -3.604 0.000314 ***
## age           0.006184   0.172556   0.036 0.971410
## mean_probsm   1.822261   0.349373   5.216 1.83e-07 ***
## mean_monitor -0.514949   0.271600  -1.896 0.057962 .
## ---
## Signif. codes:  0 '***' 0.001 '**' 0.01 '*' 0.05 '.' 0.1 ' ' 1
##
## Correlation of Fixed Effects:
##             (Intr) sexFml age    mn_prb
## sexFemale    0.003
## age         -0.907  0.032
## mean_probsm -0.316 -0.260  0.006
## mean_monitr -0.298  0.055  0.123 -0.196
```

```r
summary(victim_primary)
```

```
## Generalized linear mixed model fit by maximum likelihood (Laplace
##   Approximation) [glmerMod]
##  Family: binomial  ( logit )
## Formula: victim ~ sex + age + mean_probsm + mean_monitor + mean_conceal +
##     (1 | school)
##    Data: df_use
## Control: ctrl
##
##       AIC       BIC    logLik -2*log(L)  df.resid
##     170.4     202.1     -78.2     156.4       679
##
## Scaled residuals:
##     Min      1Q  Median      3Q     Max
## -1.1137 -0.1697 -0.0981 -0.0550  8.2603
##
## Random effects:
##  Groups Name        Variance Std.Dev.
##  school (Intercept) 0.3347   0.5786
## Number of obs: 686, groups:  school, 12
##
## Fixed effects:
##              Estimate Std. Error z value Pr(>|z|)
## (Intercept)  -6.67840    3.03293  -2.202 0.027668 *
## sexFemale    -1.78449    0.51985  -3.433 0.000598 ***
## age          -0.01609    0.17592  -0.091 0.927120
## mean_probsm   1.45030    0.37242   3.894 9.85e-05 ***
## mean_monitor -0.40224    0.27496  -1.463 0.143491
## mean_conceal  0.56888    0.25851   2.201 0.027761 *
## ---
## Signif. codes:  0 '***' 0.001 '**' 0.01 '*' 0.05 '.' 0.1 ' ' 1
##
```

```
## Correlation of Fixed Effects:
##             (Intr) sexFml age    mn_prb mn_mnt
## sexFemale   -0.058
## age         -0.907  0.089
## mean_probsm -0.239 -0.240  0.021
## mean_monitr -0.304  0.088  0.117 -0.255
## mean_concel -0.053 -0.006 -0.069 -0.403  0.150
```

9.2 Perpetration

```r
agg_conceal <- lme4::glmer(
  agg ~ sex + age + mean_probsm + mean_conceal + (1 | school),
  data = df_use,
  family = binomial(link = "logit")
)

agg_monitor <- lme4::glmer(
  agg ~ sex + age + mean_probsm + mean_monitor + (1 | school),
  data = df_use,
  family = binomial(link = "logit")
)

summary(agg_conceal)
```

```
## Generalized linear mixed model fit by maximum likelihood (Laplace
##   Approximation) [glmerMod]
##  Family: binomial  ( logit )
## Formula: agg ~ sex + age + mean_probsm + mean_conceal + (1 | school)
##    Data: df_use
##
##       AIC       BIC    logLik -2*log(L)  df.resid
##     342.3     369.5    -165.2     330.3       680
##
## Scaled residuals:
##     Min      1Q  Median      3Q     Max
## -1.0848 -0.2904 -0.2231 -0.1655 10.1917
##
## Random effects:
##  Groups Name        Variance Std.Dev.
##  school (Intercept) 0.2362   0.486
## Number of obs: 686, groups:  school, 12
##
## Fixed effects:
##              Estimate Std. Error z value Pr(>|z|)
## (Intercept)   -6.5019     2.1014  -3.094  0.00197 **
## sexFemale     -0.5203     0.3109  -1.673  0.09427 .
## age            0.1017     0.1294   0.786  0.43191
## mean_probsm    0.6676     0.2279   2.929  0.00340 **
## mean_conceal   0.4220     0.1789   2.358  0.01836 *
## ---
## Signif. codes:  0 '***' 0.001 '**' 0.01 '*' 0.05 '.' 0.1 ' ' 1
##
## Correlation of Fixed Effects:
##             (Intr) sexFml age    mn_prb
## sexFemale   -0.081
## age         -0.952  0.052
## mean_probsm -0.247 -0.204  0.041
## mean_concel -0.025  0.096 -0.060 -0.414
```

```r
summary(agg_monitor)
```

```
## Generalized linear mixed model fit by maximum likelihood (Laplace
##   Approximation) [glmerMod]
##  Family: binomial  ( logit )
## Formula: agg ~ sex + age + mean_probsm + mean_monitor + (1 | school)
##    Data: df_use
##
##       AIC       BIC    logLik -2*log(L)  df.resid
##     347.4     374.5    -167.7     335.4       680
##
## Scaled residuals:
##     Min      1Q  Median      3Q     Max
## -0.8413 -0.2991 -0.2308 -0.1641  9.6362
##
## Random effects:
##  Groups Name        Variance Std.Dev.
##  school (Intercept) 0.2763   0.5256
## Number of obs: 686, groups:  school, 12
##
## Fixed effects:
##              Estimate Std. Error z value Pr(>|z|)
## (Intercept)  -6.04003    2.24386  -2.692  0.00711 **
## sexFemale    -0.58405    0.30663  -1.905  0.05682 .
## age           0.11053    0.12904   0.857  0.39169
## mean_probsm   0.89705    0.20935   4.285 1.83e-05 ***
## mean_monitor -0.08165    0.17427  -0.469  0.63940
## ---
## Signif. codes:  0 '***' 0.001 '**' 0.01 '*' 0.05 '.' 0.1 ' ' 1
##
## Correlation of Fixed Effects:
##             (Intr) sexFml age    mn_prb
## sexFemale   -0.031
## age         -0.931  0.029
## mean_probsm -0.265 -0.189  0.016
## mean_monitr -0.380 -0.058  0.160 -0.009
```

```r
summary(agg_primary)
```

```
## Generalized linear mixed model fit by maximum likelihood (Laplace
##   Approximation) [glmerMod]
##  Family: binomial  ( logit )
## Formula: agg ~ sex + age + mean_probsm + mean_monitor + mean_conceal +
##     (1 | school)
##    Data: df_use
## Control: glmerControl(optimizer = "bobyqa", optCtrl = list(maxfun = 1e+05))
##
##       AIC       BIC    logLik -2*log(L)  df.resid
##     344.3     376.0    -165.1     330.3       679
##
## Scaled residuals:
##     Min      1Q  Median      3Q     Max
```

```
## -1.0823 -0.2905 -0.2228 -0.1661 10.2111
##
## Random effects:
##  Groups Name        Variance Std.Dev.
##  school (Intercept) 0.2375   0.4873
## Number of obs: 686, groups:  school, 12
##
## Fixed effects:
##              Estimate Std. Error z value Pr(>|z|)
## (Intercept)   -6.5596     2.2788  -2.879  0.00400 **
## sexFemale     -0.5207     0.3110  -1.674  0.09408 .
## age            0.1029     0.1308   0.787  0.43132
## mean_probsm    0.6660     0.2292   2.906  0.00366 **
## mean_monitor   0.0118     0.1793   0.066  0.94754
## mean_conceal   0.4248     0.1838   2.311  0.02083 *
## ---
## Signif. codes:  0 '***' 0.001 '**' 0.01 '*' 0.05 '.' 0.1 ' ' 1
##
## Correlation of Fixed Effects:
##             (Intr) sexFml age    mn_prb mn_mnt
## sexFemale   -0.066
## age         -0.925  0.048
## mean_probsm -0.186 -0.200  0.026
## mean_monitr -0.386 -0.023  0.142 -0.106
## mean_concel -0.110  0.088 -0.026 -0.425  0.230
```

10 Sensitivity Analyses with Additional Covariates

Perceived family financial resources are treated as an ordered numeric covariate from 1 to 4. Responses indicating uncertainty are treated as missing. The base and fully adjusted models are estimated in the same complete-case sample.

```
# Convert substantive financial-resource responses to numeric values.
df_use <- df_use %>%
  dplyr::mutate(
    money_num = as.numeric(as.character(money)),
    money_num = ifelse(money_num == 5, NA, money_num)
  )

df_sens <- df_use %>%
  dplyr::filter(
    complete.cases(
      victim, agg, sex, age, school,
      mean_probsm, mean_monitor, mean_conceal,
      white, money_num
    )
  )

nrow(df_sens)
```

```
## [1] 555
```

```
table(df_sens$white)
```

```
##
##  No Yes
## 115 440
```

```
table(df_sens$money_num)
```

```
##
##   1   2   3   4
##  11  75 234 235
```

10.1 Victimization

```
victim_base_sens <- lme4::glmer(
  victim ~ sex + age + mean_probsm + mean_monitor + mean_conceal +
    (1 | school),
  data = df_sens,
  family = binomial(link = "logit"),
  control = glmerControl(optimizer = "Nelder_Mead")
)

victim_full_sens_start <- lme4::glmer(
  victim ~ sex + age + white + money_num +
    mean_probsm + mean_monitor + mean_conceal +
    (1 | school),
  data = df_sens,
  family = binomial(link = "logit"),
  control = glmerControl(optimizer = "Nelder_Mead")
)

# Optimizer check for the fully adjusted victimization model.
victim_full_sens_allfit <- lme4::allFit(victim_full_sens_start)
```

```
## bobyqa : [OK]
## Nelder_Mead : [OK]
## nlminbwrap : [OK]
## optimx.L-BFGS-B : [OK]
## nloptwrap.NLOPT_LN_NELDERMEAD :

## [OK]
## nloptwrap.NLOPT_LN_BOBYQA :

## [OK]
```

```
victim_full_sens <- victim_full_sens_allfit$bobyqa

summary(victim_base_sens)
```

```
## Generalized linear mixed model fit by maximum likelihood (Laplace
##   Approximation) [glmerMod]
##  Family: binomial  ( logit )
## Formula: victim ~ sex + age + mean_probsm + mean_monitor + mean_conceal +
##     (1 | school)
##    Data: df_sens
## Control: glmerControl(optimizer = "Nelder_Mead")
##
##       AIC       BIC    logLik -2*log(L)  df.resid
##     146.9     177.1     -66.4     132.9       548
##
## Scaled residuals:
##     Min      1Q  Median      3Q     Max
## -1.0901 -0.1853 -0.1096 -0.0603  8.7326
```

```
##
## Random effects:
##  Groups Name        Variance Std.Dev.
##  school (Intercept) 0.02635  0.1623
## Number of obs: 555, groups:  school, 12
##
## Fixed effects:
##              Estimate Std. Error z value Pr(>|z|)
## (Intercept)  -5.68001    3.15158  -1.802 0.071502 .
## sexFemale    -1.70111    0.56083  -3.033 0.002420 **
## age          -0.06943    0.17594  -0.395 0.693111
## mean_probsm   1.40204    0.40703   3.445 0.000572 ***
## mean_monitor -0.36134    0.30723  -1.176 0.239548
## mean_conceal  0.56887    0.26730   2.128 0.033323 *
## ---
## Signif. codes:  0 '***' 0.001 '**' 0.01 '*' 0.05 '.' 0.1 ' ' 1
##
## Correlation of Fixed Effects:
##             (Intr) sexFml age    mn_prb mn_mnt
## sexFemale   -0.102
## age         -0.898  0.171
## mean_probsm -0.144 -0.355 -0.104
## mean_monitr -0.454  0.095  0.241 -0.193
## mean_concel -0.183  0.023  0.070 -0.409  0.221
```

```r
summary(victim_full_sens)
```

```
## Generalized linear mixed model fit by maximum likelihood (Laplace
##   Approximation) [glmerMod]
##  Family: binomial  ( logit )
## Formula: victim ~ sex + age + white + money_num + mean_probsm + mean_monitor +
##     mean_conceal + (1 | school)
##    Data: df_sens
## Control: ctrl
##
##       AIC       BIC    logLik -2*log(L)  df.resid
##     150.6     189.5     -66.3     132.6       546
##
## Scaled residuals:
##     Min      1Q  Median      3Q     Max
## -1.0670 -0.1855 -0.1077 -0.0599  9.3719
##
## Random effects:
##  Groups Name        Variance Std.Dev.
##  school (Intercept) 0.05827  0.2414
## Number of obs: 555, groups:  school, 12
##
## Fixed effects:
##              Estimate Std. Error z value Pr(>|z|)
## (Intercept)  -5.32294    3.20319  -1.662 0.096560 .
## sexFemale    -1.71308    0.56476  -3.033 0.002419 **
## age          -0.05975    0.17642  -0.339 0.734847
## whiteYes     -0.12908    0.56956  -0.227 0.820713
## money_num    -0.16138    0.33830  -0.477 0.633342
```

```
## mean_probsm   1.38652    0.41095   3.374 0.000741 ***
## mean_monitor -0.31369    0.31998  -0.980 0.326910
## mean_conceal  0.55631    0.26840   2.073 0.038198 *
## ---
## Signif. codes:  0 '***' 0.001 '**' 0.01 '*' 0.05 '.' 0.1 ' ' 1
##
## Correlation of Fixed Effects:
##              (Intr) sexFml age    whitYs mny_nm mn_prb mn_mnt
## sexFemale   -0.109
## age         -0.855  0.169
## whiteYes    -0.115  0.071  0.048
## money_num   -0.148  0.013 -0.129 -0.114
## mean_probsm -0.162 -0.354 -0.100 -0.007  0.057
## mean_monitr -0.365  0.077  0.237 -0.129 -0.205 -0.201
## mean_concel -0.182  0.034  0.052  0.084  0.043 -0.406  0.186
```

```r
lme4::isSingular(victim_base_sens)
```

```
## [1] FALSE
```

```r
lme4::isSingular(victim_full_sens)
```

```
## [1] FALSE
```

```r
anova(victim_base_sens, victim_full_sens, test = "Chisq")
```

```
## Data: df_sens
## Models:
## victim_base_sens: victim ~ sex + age + mean_probsm + mean_monitor + mean_conceal + (1 | school)
## victim_full_sens: victim ~ sex + age + white + money_num + mean_probsm + mean_monitor + mean_conceal
##                  npar    AIC    BIC  logLik -2*log(L)  Chisq Df Pr(>Chisq)
## victim_base_sens    7 146.89 177.12 -66.446    132.89
## victim_full_sens    9 150.58 189.46 -66.292    132.58 0.3077  2     0.8574
```

10.2 Perpetration

```
agg_base_sens <- lme4::glmer(
  agg ~ sex + age + mean_probsm + mean_monitor + mean_conceal +
    (1 | school),
  data = df_sens,
  family = binomial(link = "logit"),
  control = glmerControl(
    optimizer = "bobyqa",
    optCtrl = list(maxfun = 100000)
  )
)

agg_full_sens <- lme4::glmer(
  agg ~ sex + age + white + money_num +
    mean_probsm + mean_monitor + mean_conceal +
    (1 | school),
  data = df_sens,
  family = binomial(link = "logit"),
  control = glmerControl(
    optimizer = "bobyqa",
    optCtrl = list(maxfun = 100000)
  )
)

summary(agg_base_sens)
```

```
## Generalized linear mixed model fit by maximum likelihood (Laplace
##   Approximation) [glmerMod]
##  Family: binomial  ( logit )
## Formula: agg ~ sex + age + mean_probsm + mean_monitor + mean_conceal +
##     (1 | school)
##    Data: df_sens
## Control: glmerControl(optimizer = "bobyqa", optCtrl = list(maxfun = 1e+05))
##
##       AIC       BIC    logLik -2*log(L)  df.resid
##     308.8     339.1    -147.4     294.8       548
##
## Scaled residuals:
##     Min      1Q  Median      3Q     Max
## -1.0147 -0.3188 -0.2420 -0.1818  9.6374
##
## Random effects:
##  Groups Name        Variance Std.Dev.
##  school (Intercept) 0.1951   0.4418
## Number of obs: 555, groups:  school, 12
##
## Fixed effects:
##              Estimate Std. Error z value Pr(>|z|)
## (Intercept)  -4.13274    2.42819  -1.702   0.0888 .
## sexFemale    -0.44197    0.33565  -1.317   0.1879
## age          -0.02165    0.14005  -0.155   0.8772
## mean_probsm   0.61380    0.24494   2.506   0.0122 *
```

```
## mean_monitor -0.06703    0.18931  -0.354   0.7233  
## mean_conceal  0.41667    0.18772   2.220   0.0264 *
## ---
## Signif. codes:  0 '***' 0.001 '**' 0.01 '*' 0.05 '.' 0.1 ' ' 1
## 
## Correlation of Fixed Effects:
##             (Intr) sexFml age    mn_prb mn_mnt
## sexFemale   -0.070                            
## age         -0.927  0.069                     
## mean_probsm -0.129 -0.283 -0.038              
## mean_monitr -0.433 -0.029  0.197 -0.097       
## mean_concel -0.131  0.113 -0.006 -0.413  0.244
```

```r
summary(agg_full_sens)
```

```
## Generalized linear mixed model fit by maximum likelihood (Laplace
##   Approximation) [glmerMod]
##  Family: binomial  ( logit )
## Formula: agg ~ sex + age + white + money_num + mean_probsm + mean_monitor +  
##     mean_conceal + (1 | school)
##    Data: df_sens
## Control: glmerControl(optimizer = "bobyqa", optCtrl = list(maxfun = 1e+05))
## 
##       AIC       BIC    logLik -2*log(L)  df.resid 
##     311.6     350.5    -146.8     293.6       546 
## 
## Scaled residuals: 
##     Min      1Q  Median      3Q     Max 
## -1.1265 -0.3178 -0.2382 -0.1834 10.1244 
## 
## Random effects:
##  Groups Name        Variance Std.Dev.
##  school (Intercept) 0.186    0.4313  
## Number of obs: 555, groups:  school, 12
## 
## Fixed effects:
##              Estimate Std. Error z value Pr(>|z|)  
## (Intercept)  -3.58299    2.50742  -1.429   0.1530  
## sexFemale    -0.43364    0.33655  -1.288   0.1976  
## age          -0.01075    0.14308  -0.075   0.9401  
## whiteYes     -0.11515    0.37646  -0.306   0.7597  
## money_num    -0.21971    0.21084  -1.042   0.2974  
## mean_probsm   0.57743    0.24711   2.337   0.0195 *
## mean_monitor -0.01463    0.19562  -0.075   0.9404  
## mean_conceal  0.40833    0.18853   2.166   0.0303 *
## ---
## Signif. codes:  0 '***' 0.001 '**' 0.01 '*' 0.05 '.' 0.1 ' ' 1
## 
## Correlation of Fixed Effects:
##             (Intr) sexFml age    whitYs mny_nm mn_prb mn_mnt
## sexFemale   -0.063                                          
## age         -0.897  0.066                                   
## whiteYes    -0.085  0.034  0.017                            
## money_num   -0.158 -0.031 -0.081 -0.091
```

```
## mean_probsm -0.160 -0.286 -0.033  0.026  0.119
## mean_monitr -0.356 -0.024  0.196 -0.133 -0.208 -0.112
## mean_concel -0.119  0.117 -0.023  0.033  0.017 -0.405  0.223
```

```r
lme4::isSingular(agg_base_sens)
```

```
## [1] FALSE
```

```r
lme4::isSingular(agg_full_sens)
```

```
## [1] FALSE
```

```r
anova(agg_base_sens, agg_full_sens, test = "Chisq")
```

```
## Data: df_sens
## Models:
## agg_base_sens: agg ~ sex + age + mean_probsm + mean_monitor + mean_conceal + (1 | school)
## agg_full_sens: agg ~ sex + age + white + money_num + mean_probsm + mean_monitor + mean_conceal + (1
##               npar    AIC    BIC  logLik -2*log(L)  Chisq Df Pr(>Chisq)
## agg_base_sens    7 308.82 339.05 -147.41    294.82
## agg_full_sens    9 311.61 350.48 -146.81    293.61 1.2125  2     0.5454
```

```r
# Recreate missingness criteria before analytic-sample filtering
check_excluded <- dfl %>%
  dplyr::mutate(
    agg = ifelse(bully_1 > 2, 1, 0),
    victim = ifelse(bully_2 > 2, 1, 0),
    mean_bully = rowmeans(bully_1, bully_2),
    risk = ifelse(mean_bully > 2, 1, 0),

    nmiss = rowsums(
      is.na(hide_1), is.na(hide_2), is.na(hide_3), is.na(hide_4),
      is.na(monitor_1), is.na(monitor_2), is.na(monitor_3), is.na(monitor_4),
      is.na(monitor_5), is.na(monitor_6), is.na(monitor_7), is.na(monitor_8),
      is.na(bully_1), is.na(bully_2),
      is.na(probsm_1), is.na(probsm_2), is.na(probsm_3), is.na(probsm_4),
      is.na(probsm_5), is.na(probsm_6), is.na(probsm_7),
      is.na(risk), is.na(agg), is.na(victim)
    )
  ) %>%

  # Restrict to adolescents who reported social media use
  dplyr::filter(smuse == 1) %>%

  # Show ONLY people who failed your final analytic-sample criteria
  dplyr::filter(nmiss >= 24 | is.na(risk)) %>%

  # Only show useful diagnostic variables
  dplyr::select(
    pid,
    smuse,
    nmiss,
    risk,
    agg,
    victim,
    bully_1,
    bully_2,
    probsm_1:probsm_7,
    hide_1:hide_4,
    monitor_1:monitor_8
  )

nrow(check_excluded)
```

```
## [1] 331
```

```r
check_excluded
```

```
## # A tibble: 331 x 27
##      pid smuse nmiss  risk   agg victim bully_1 bully_2 probsm_1 probsm_2
##    <dbl> <dbl> <dbl> <dbl> <dbl>  <dbl>   <dbl>   <dbl>    <dbl>    <dbl>
##  1   156     1    24    NA    NA     NA      NA      NA       NA       NA
##  2   122     1    24    NA    NA     NA      NA      NA       NA       NA
##  3   253     1    24    NA    NA     NA      NA      NA       NA       NA
##  4   292     1    24    NA    NA     NA      NA      NA       NA       NA
```

```
##  5   189     1    24    NA    NA     NA      NA      NA       NA       NA
##  6   139     1    24    NA    NA     NA      NA      NA       NA       NA
##  7   127     1    24    NA    NA     NA      NA      NA       NA       NA
##  8   123     1    24    NA    NA     NA      NA      NA       NA       NA
##  9   114     1    24    NA    NA     NA      NA      NA       NA       NA
## 10   151     1    24    NA    NA     NA      NA      NA       NA       NA
## # i 321 more rows
## # i 17 more variables: probsm_3 <dbl>, probsm_4 <dbl>, probsm_5 <dbl>,
## #   probsm_6 <dbl>, probsm_7 <dbl>, hide_1 <dbl>, hide_2 <dbl>, hide_3 <dbl>,
## #   hide_4 <dbl>, monitor_1 <dbl>, monitor_2 <dbl>, monitor_3 <dbl>,
## #   monitor_4 <dbl>, monitor_5 <dbl>, monitor_6 <dbl>, monitor_7 <dbl>,
## #   monitor_8 <dbl>
```

```
# Identify the 7 social media users excluded from the final analytic sample

check_excluded <- dfl %>%
  dplyr::mutate(

    # Recreate cyberbullying variables used in the analytic-sample cleaning
    agg = ifelse(bully_1 > 2, 1, 0),
    victim = ifelse(bully_2 > 2, 1, 0),
    mean_bully = rowmeans(bully_1, bully_2),
    risk = ifelse(mean_bully > 2, 1, 0),

    # Recreate the missing-data count used in the original cleaning
    nmiss = rowsums(
      is.na(hide_1), is.na(hide_2), is.na(hide_3), is.na(hide_4),
      is.na(monitor_1), is.na(monitor_2), is.na(monitor_3), is.na(monitor_4),
      is.na(monitor_5), is.na(monitor_6), is.na(monitor_7), is.na(monitor_8),
      is.na(bully_1), is.na(bully_2),
      is.na(probsm_1), is.na(probsm_2), is.na(probsm_3), is.na(probsm_4),
      is.na(probsm_5), is.na(probsm_6), is.na(probsm_7),
      is.na(risk), is.na(agg), is.na(victim)
    )
  ) %>%

  # smuse = 2 identifies the 693 adolescents who reported social media use
  dplyr::filter(smuse == 2) %>%

  # These are the exact two exclusion criteria used to create df_use
  dplyr::filter(nmiss >= 24 | is.na(risk)) %>%

  # Show only variables useful for determining why they were excluded
  dplyr::select(
    pid,
    smuse,
    nmiss,
    risk,
    agg,
    victim,
    bully_1,
    bully_2,
    probsm_1:probsm_7,
    hide_1:hide_4,
```

```r
    monitor_1:monitor_8
  )

# Number excluded
nrow(check_excluded)
```

```
## [1] 7
```

```r
# View only those excluded participants
check_excluded
```

```
## # A tibble: 7 x 27
##     pid smuse nmiss  risk   agg victim bully_1 bully_2 probsm_1 probsm_2
##   <dbl> <dbl> <dbl> <dbl> <dbl>  <dbl>   <dbl>   <dbl>    <dbl>    <dbl>
## 1   102     2    13    NA    NA     NA      NA      NA        2        2
## 2   577     2    24    NA    NA     NA      NA      NA       NA       NA
## 3   449     2    13    NA    NA     NA      NA      NA        3        4
## 4  3049     2     6    NA     0     NA       2      NA        3        3
## 5  1461     2    24    NA    NA     NA      NA      NA       NA       NA
## 6   723     2     5    NA    NA     NA      NA      NA        4        2
## 7   725     2    13    NA    NA     NA      NA      NA        4        3
## # i 17 more variables: probsm_3 <dbl>, probsm_4 <dbl>, probsm_5 <dbl>,
## #   probsm_6 <dbl>, probsm_7 <dbl>, hide_1 <dbl>, hide_2 <dbl>, hide_3 <dbl>,
## #   hide_4 <dbl>, monitor_1 <dbl>, monitor_2 <dbl>, monitor_3 <dbl>,
## #   monitor_4 <dbl>, monitor_5 <dbl>, monitor_6 <dbl>, monitor_7 <dbl>,
## #   monitor_8 <dbl>
```

```r
check_excluded %>%
  dplyr::select(
    pid,
    nmiss,
    risk,
    agg,
    victim,
    bully_1,
    bully_2
  )
```

```
## # A tibble: 7 x 7
##     pid nmiss  risk   agg victim bully_1 bully_2
##   <dbl> <dbl> <dbl> <dbl>  <dbl>   <dbl>   <dbl>
## 1   102    13    NA    NA     NA      NA      NA
## 2   577    24    NA    NA     NA      NA      NA
## 3   449    13    NA    NA     NA      NA      NA
## 4  3049     6    NA     0     NA       2      NA
## 5  1461    24    NA    NA     NA      NA      NA
## 6   723     5    NA    NA     NA      NA      NA
## 7   725    13    NA    NA     NA      NA      NA
```

```r
check_excluded %>%
  dplyr::count(nmiss)
```

```
## # A tibble: 4 x 2
##   nmiss     n
##   <dbl> <int>
## 1     5     1
## 2     6     1
## 3    13     3
## 4    24     2
```

```r
table(df_use$sex, useNA = "ifany")
```

```
##
##   Male Female
##    280    406
```

```r
sum(!is.na(df_use$sex))
```

```
## [1] 686
```

```r
sum(is.na(df_use$sex))
```

```
## [1] 0
```

```r
nrow(df_use)
```

```
## [1] 686
```

```r
nobs(victim_primary)
```

```
## [1] 686
```

```r
nobs(agg_primary)
```

```
## [1] 686
```

11 Session Information

```r
sessionInfo()
```

```
## R version 4.5.2 (2025-10-31 ucrt)
## Platform: x86_64-w64-mingw32/x64
## Running under: Windows 11 x64 (build 26200)
##
## Matrix products: default
##   LAPACK version 3.12.1
##
## locale:
```

```
## [1] LC_COLLATE=English_United States.utf8
## [2] LC_CTYPE=English_United States.utf8
## [3] LC_MONETARY=English_United States.utf8
## [4] LC_NUMERIC=C
## [5] LC_TIME=English_United States.utf8
##
## time zone: America/Denver
## tzcode source: internal
##
## attached base packages:
## [1] stats     graphics  grDevices utils     datasets  methods   base
##
## other attached packages:
##  [1] car_3.1-5          carData_3.0-6      performance_0.16.0 lme4_1.1-38
##  [5] Matrix_1.7-4       furniture_1.11.0   lubridate_1.9.5    forcats_1.0.1
##  [9] stringr_1.6.0      dplyr_1.2.0        purrr_1.2.1        readr_2.1.6
## [13] tidyr_1.3.2        tibble_3.3.1       ggplot2_4.0.2      tidyverse_2.0.0
##
## loaded via a namespace (and not attached):
##  [1] tidyselect_1.2.1       psych_2.6.1            farver_2.1.2
##  [4] S7_0.2.1               fastmap_1.2.0          pracma_2.4.6
##  [7] fontquiver_0.2.1       digest_0.6.39          timechange_0.4.0
## [10] lifecycle_1.0.5        magrittr_2.0.4         compiler_4.5.2
## [13] rlang_1.1.7            tools_4.5.2            yaml_2.3.12
## [16] gt_1.3.0               data.table_1.18.2.1    knitr_1.51
## [19] askpass_1.2.1          bit_4.6.0              mnormt_2.1.2
## [22] xml2_1.5.2             RColorBrewer_1.1-3     abind_1.4-8
## [25] numDeriv_2016.8-1.1    withr_3.0.2            grid_4.5.2
## [28] gdtools_0.4.4          scales_1.4.0           MASS_7.3-65
## [31] insight_1.4.6          cli_3.6.5              rmarkdown_2.30
## [34] crayon_1.5.3           ragg_1.5.0             reformulas_0.4.4
## [37] generics_0.1.4         otel_0.2.0             rstudioapi_0.18.0
## [40] tzdb_0.5.0             minqa_1.2.8            splines_4.5.2
## [43] parallel_4.5.2         vctrs_0.7.1            boot_1.3-32
## [46] fontBitstreamVera_0.1.1 hms_1.1.4             bit64_4.6.0-1
## [49] Formula_1.2-5          systemfonts_1.3.1      optimx_2025-4.9
## [52] glue_1.8.0             nloptr_2.2.1           stringi_1.8.7
## [55] flextable_0.9.10       gtable_0.3.6           pillar_1.11.1
## [58] htmltools_0.5.9        openssl_2.3.4          R6_2.6.1
## [61] textshaping_1.0.4      Rdpack_2.6.5           vroom_1.7.0
## [64] evaluate_1.0.5         lattice_0.22-7         rbibutils_2.4.1
## [67] fontLiberation_0.1.0   Rcpp_1.1.1             zip_2.3.3
## [70] uuid_1.2-2             nlme_3.1-168           officer_0.7.3
## [73] xfun_0.56              fs_1.6.6               pkgconfig_2.0.3
```
